# A Highly Conductive *n*-Type Conjugated Polymer Synthesized in Water

**DOI:** 10.1021/jacs.4c02270

**Published:** 2024-05-30

**Authors:** Qifan Li, Jun-Da Huang, Tiefeng Liu, Tom P. A. van der Pol, Qilun Zhang, Sang Young Jeong, Marc-Antoine Stoeckel, Han-Yan Wu, Silan Zhang, Xianjie Liu, Han Young Woo, Mats Fahlman, Chi-Yuan Yang, Simone Fabiano

**Affiliations:** † Laboratory of Organic Electronics, Department of Science and Technology, Linköping University, SE-60174 Norrköping, Sweden; ‡ Wallenberg Wood Science Center, Department of Science and Technology, Linköping University, SE-60174 Norrköping, Sweden; § Wallenberg Initiative Materials Science for Sustainability, Department of Science and Technology, Linköping University, SE-60174 Norrköping, Sweden; ∥ Department of Chemistry, College of Science, Korea University, Seoul 136-713, Republic of Korea; ⊥ n-Ink AB, Bredgatan 33, SE-60221 Norrköping, Sweden

## Abstract

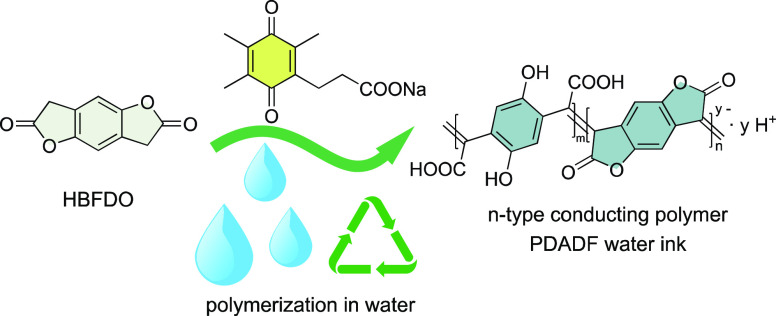

Poly(3,4-ethylenedioxythiophene):poly(styrenesulfonate) (PEDOT:PSS) is a benchmark hole-transporting (*p*-type) polymer that finds applications in diverse electronic devices. Most of its success is due to its facile synthesis in water, exceptional processability from aqueous solutions, and outstanding electrical performance in ambient. Applications in fields like (opto-)electronics, bioelectronics, and energy harvesting/storage devices often necessitate the complementary use of both *p*-type and *n*-type (electron-transporting) materials. However, the availability of *n*-type materials amenable to water-based polymerization and processing remains limited. Herein, we present a novel synthesis method enabling direct polymerization in water, yielding a highly conductive, water-processable *n*-type conjugated polymer, namely, poly[(2,2′-(2,5-dihydroxy-1,4-phenylene)diacetic acid)-*stat*-3,7-dihydrobenzo[1,2-b:4,5-b′]difuran-2,6-dione] (PDADF), with remarkable electrical conductivity as high as 66 S cm^–1^, ranking among the highest for *n*-type polymers processed using green solvents. The new *n*-type polymer PDADF also exhibits outstanding stability, maintaining 90% of its initial conductivity after 146 days of storage in air. Our synthetic approach, along with the novel polymer it yields, promises significant advancements for the sustainable development of organic electronic materials and devices.

## Introduction

**Figure 1:**
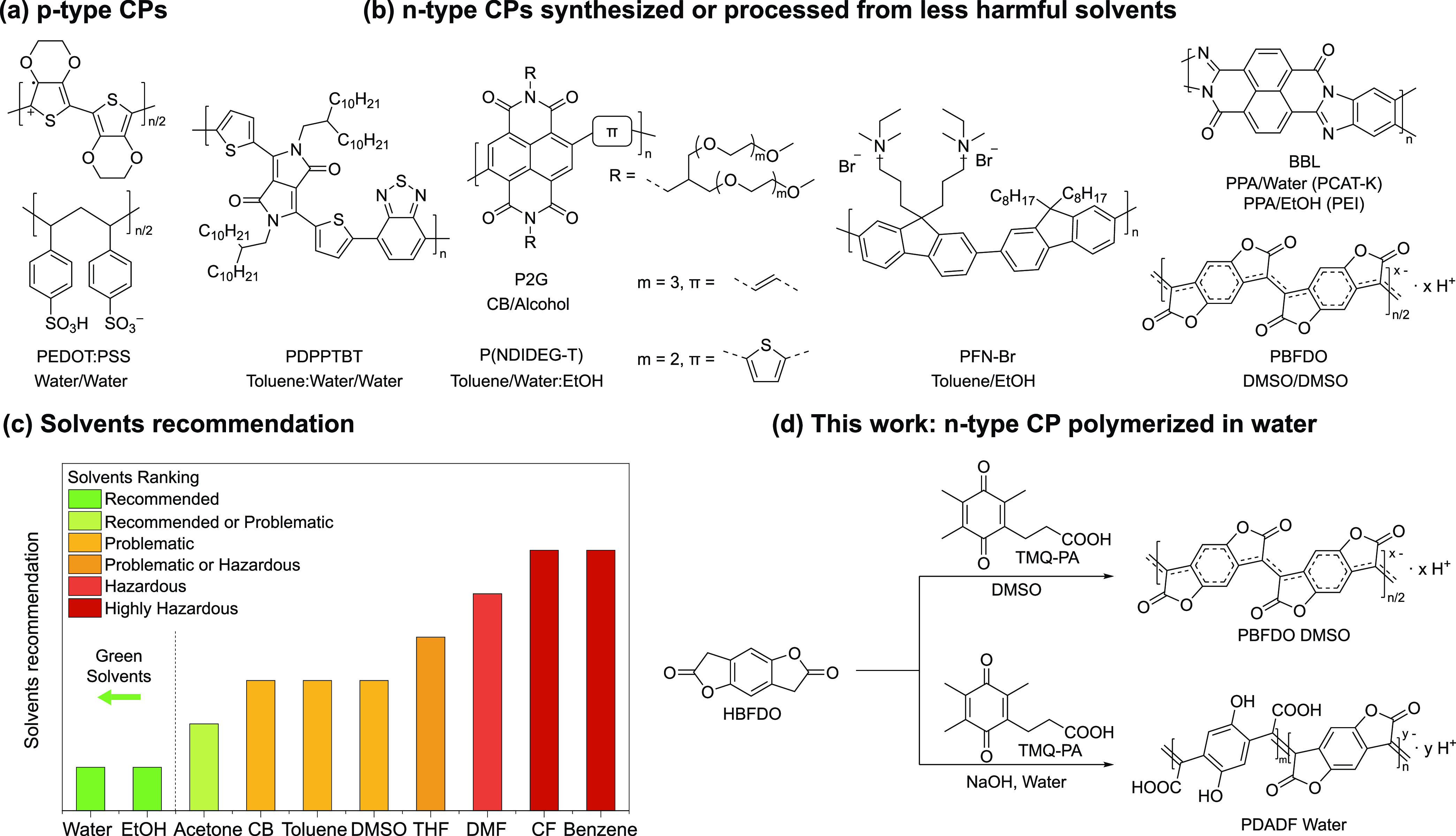
Chemical structure of representative *p*-type (a) and *n*-type conducting polymers (b). Solvents used during synthesis/processing are specified. (c) Solvent recommendations. (d) Schematic diagram for the synthesis of PBFDO and PDADF using the TMQ-PA catalyst.

Conducting polymers continue to attract significant interest owing to their robust mechanical characteristics, cost-effective solution processability over large areas, and the ease with which their physical and chemical properties can be tailored through monomer modifications.^1−4^ These attributes render conducting polymers suitable for diverse applications, including bioelectronics,^5−10^ electronic skin,^11^ biosensors,^12^ and energy harvesting/storage.^13^ A notable example of a commercially available conducting polymer is the hole-transporting (*p*-type) polymer poly(3,4-ethylenedioxythiophene):poly(styrenesulfonate) (PEDOT:PSS, Figure 1a),^14^ featuring electrical conductivity exceeding 1000 S cm^–1^ upon secondary doping.^15,16^

PEDOT:PSS is synthesized from an aqueous PSS solution through the addition of monomers and oxidants under ambient conditions (Figure S1a).^14^ The ease of its synthesis, coupled with low production costs and the use of environmentally benign solvents like water, has propelled PEDOT:PSS to the forefront of the organic electronics market. It finds diverse applications in light-emitting diodes,^17^ electronic textiles,^18^ battery/supercapacitors,^19^ electrochemical transistors,^20^ and thermoelectric generators,^21,22^ to name a few. Nevertheless, to achieve superior performance in many of these applications, it is necessary to incorporate both *p*-type and *n*-type (electron-transporting) materials.

While the development of high-performance *n*-type conducting polymers has made significant strides in recent years, it often entails the use of toxic organotin reagents, nonrecyclable precious metal catalysts (e.g., Pd), and the use of harmful solvents like chlorobenzene, tetrahydrofuran, and dimethylformamide (Figure S1b–e).^23−29^ Furthermore, these challenges extend beyond the synthetic stage, with many *n*-type conducting polymers requiring flammable or hazardous solvents during processing.^30,31^ These limitations have constrained their potential for commercial and industrial deployment.

Several notable pioneering efforts have been made to make the synthesis and processing of *n*-type conducting polymers more sustainable. For example, mini-emulsion polymerizations have been employed for the synthesis of diketopyrrolopyrrole (DPP)-based polymers via Suzuki-Miyaura coupling in a toluene:water mixture, albeit still using Pd as the catalyst.^32^ Notably, several water/alcohol-processable DPP-,^33^ naphthalenediimide-,^34,35^ and fluorene-based polymers^36−39^ (Figure 1b) have recently been reported, although the use of harmful solvents (e.g., THF and chlorobenzene) or toxic organotin catalysts in their lengthy synthetics steps remains a concern. In addition, all these works resulted in the development of polymers with low electrical conductivity (<10^–3^ S cm^–1^). The use of nonconjugated^40^ or conjugated^41,42^ polymeric dopants enabled the processing of alcohol- or water-based conductive inks with electrical conductivity in the range of 2–8 S cm^–1^ (Figure 1b).

Recently, Tang and co-workers reported on the synthesis of poly(3,7-dihydrobenzo[1,2-b:4,5-b′]difuran-2,6-dione) (PBFDO) in dimethyl sulfoxide (DMSO), demonstrating unprecedented electrical conductivities exceeding 1000 S cm^–1^ for an *n*-type polymer.^43,44^ Despite this remarkable conductivity, the purification steps of PBFDO necessitate substantial amounts of DMSO during the dialysis process. While this work marks a first step toward developing *n*-type conducting polymers processed with benign solvents, the reliance on DMSO, a solvent known for its adverse health effects,^45−47^ underscores the need for alternative approaches. Water, being the safest and most sustainable option^48^ (Figure 1c), continues to be the preferred choice for the synthesis and processing of conductive inks for large-scale printed electronics.

Here, we report the synthesis in water of the conducting copolymer poly[(2,2′-(2,5-dihydroxy-1,4-phenylene)diacetic acid)-*stat*-3,7-dihydrobenzo[1,2-b:4,5-b′]difuran-2,6-dione] (PDADF, Figure 1d). To achieve this, we designed the water-soluble organic catalyst sodium 3-(2,4,5-trimethyl-3,6-dioxocyclohexa-1,4-dien-1-yl)propanoate (TMQ-PANa), enabling the polymerization of 3,7-dihydrobenzo[1,2-b:4,5-b′]difuran-2,6-dione (HBFDO) in pure water and allowing for the recovery of the catalyst postreaction. This stands in contrast to the work of Tang et al.,^43^ where the consumption of duroquinone (TMQ) during the oxidative polymerization of HBFDO limits catalyst recovery in the synthesis of PBFDO. This novel approach results in the successful production of PDADF, showcasing remarkable electrical conductivity exceeding 48 ± 18 S cm^–1^ (max 66 S cm^–1^), among the highest reported for *n*-type polymers synthesized and processed in water. Notably, the spin-cast film demonstrates outstanding air stability, retaining 90% of the initial conductivity after 146 days in ambient without encapsulation. To illustrate the practical utility of PDADF, we demonstrate its application as the *n*-type component in thermoelectric generators (TEGs), achieving power outputs of about 15 nW at a temperature difference of 50 K.

## Results and Discussion

### Catalyst Design and Polymer Synthesis

**Figure 2:**
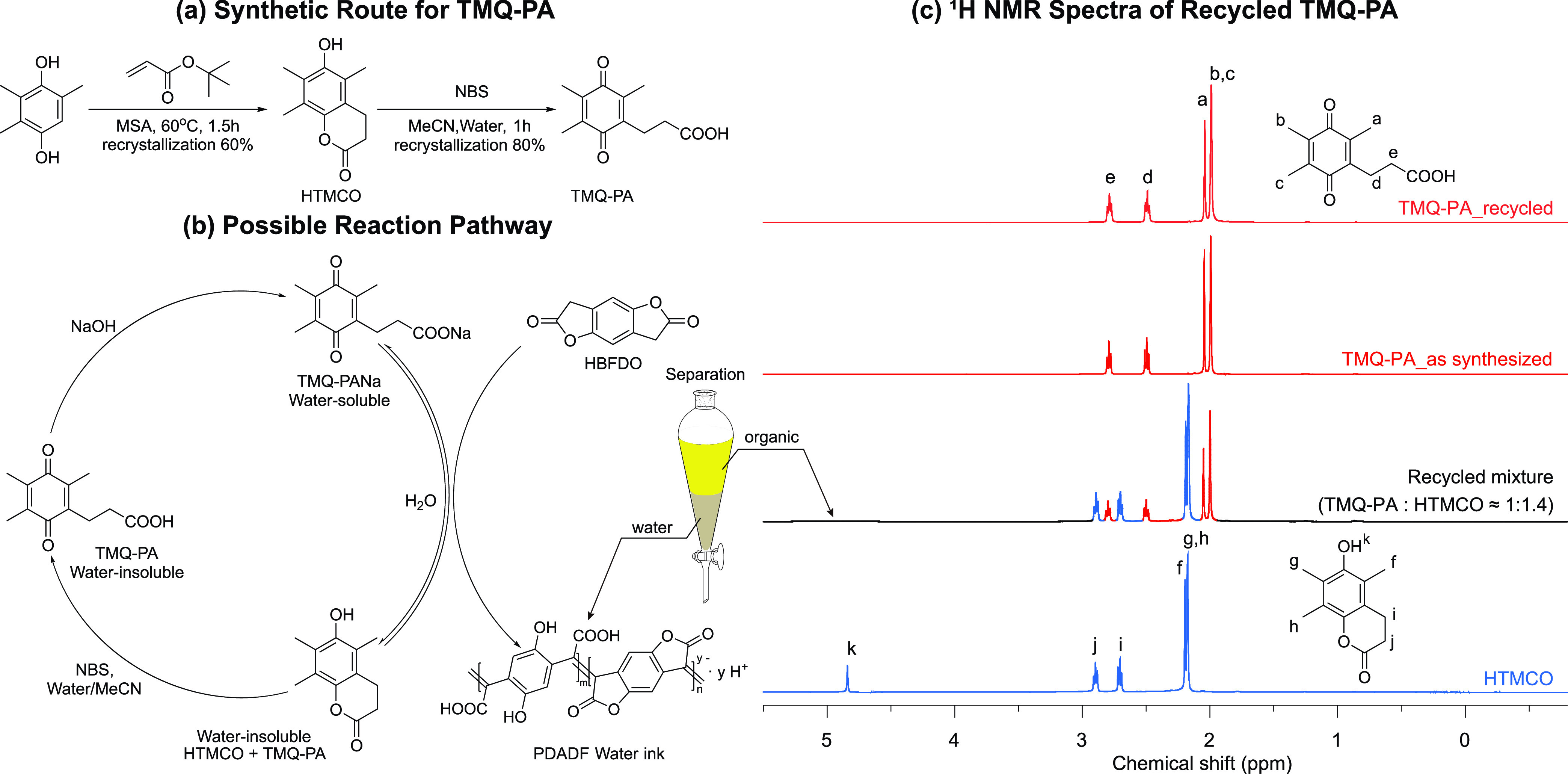
(a) Synthetic route for TMQ-PA. (b) Possible reaction pathway for the polymerization of PDADF and catalyst recycling. (c) ^1^H NMR spectra of the recycled catalyst.

Attempts to polymerize HBFDO directly from water using duroquinone (TMQ)^43^ as the oxidant failed because of the poor solubility of TMQ in water, which yields the formation of a reddish brown mixture and no polymerization (Figure S2b). To address this issue, we introduced a carboxyl group to TMQ to enhance its water solubility using the synthetic approach reported in Figure 2a. TMQ-PA (3-(2,4,5-trimethyl-3,6-dioxocyclohexa-1,4-dien-1-yl)propanoic acid) was synthesized in two steps with an overall yield of 48% (see details in the Supporting Information).^49^

First, we tested the catalytic efficiency of TMQ-PA to oxidize HBFDO and compared it to TMQ by synthesizing PBFDO in DMSO following the procedure outlined by Tang and co-workers (Figure S2c). The Fourier-transformed infrared (FTIR) spectra of PBFDO produced by TMQ-PA and TMQ exhibit identical absorption features, including the characteristic carbonyl peak at 1781 cm^–1^, along with an indistinguishable fingerprint region (Figure S3). However, attempts to synthesize PBFDO by reacting TMQ-PA with HBFDO in water were unsuccessful due to the still limited solubility of TMQ-PA in water (Figure S2d). This issue was resolved by introducing 1 eq sodium hydroxide (NaOH), resulting in the formation of the water-soluble TMQ-PANa and the complete dissolution of TMQ-PA in water. The addition of 1 eq of HBFDO to the aqueous TMQ-PANa solution resulted in the formation of PDADF, shown in Figure 2b as a partially ring-opened PBFDO copolymer structure (vide infra). Notably, upon reaction with HBFDO, TMQ-PANa precipitates as the water-insoluble 6-hydroxy-5,7,8-trimethylchroman-2-one (HTMCO) and can be recovered by solvent extraction using a mixture of water and diethyl ether. Proton nuclear magnetic resonance (^1^H NMR) performed on the extracted compounds revealed a TMQ-PA:HTMCO ratio of approximately 1:1.4 (Figure 2c). HTMCO can be reoxidized to TMQ-PA using the same synthetic procedure in Figure 2a, allowing for a 74% recovery of the initial oxidant with purity >99%.^49^ The calculated E-factor^50^ for the synthesis of PDADF is >40× smaller than that of PBFDO and other prominent *n*-type (semi)conductors (Figure S4).

### Polymer Characterization

**Figure 3:**
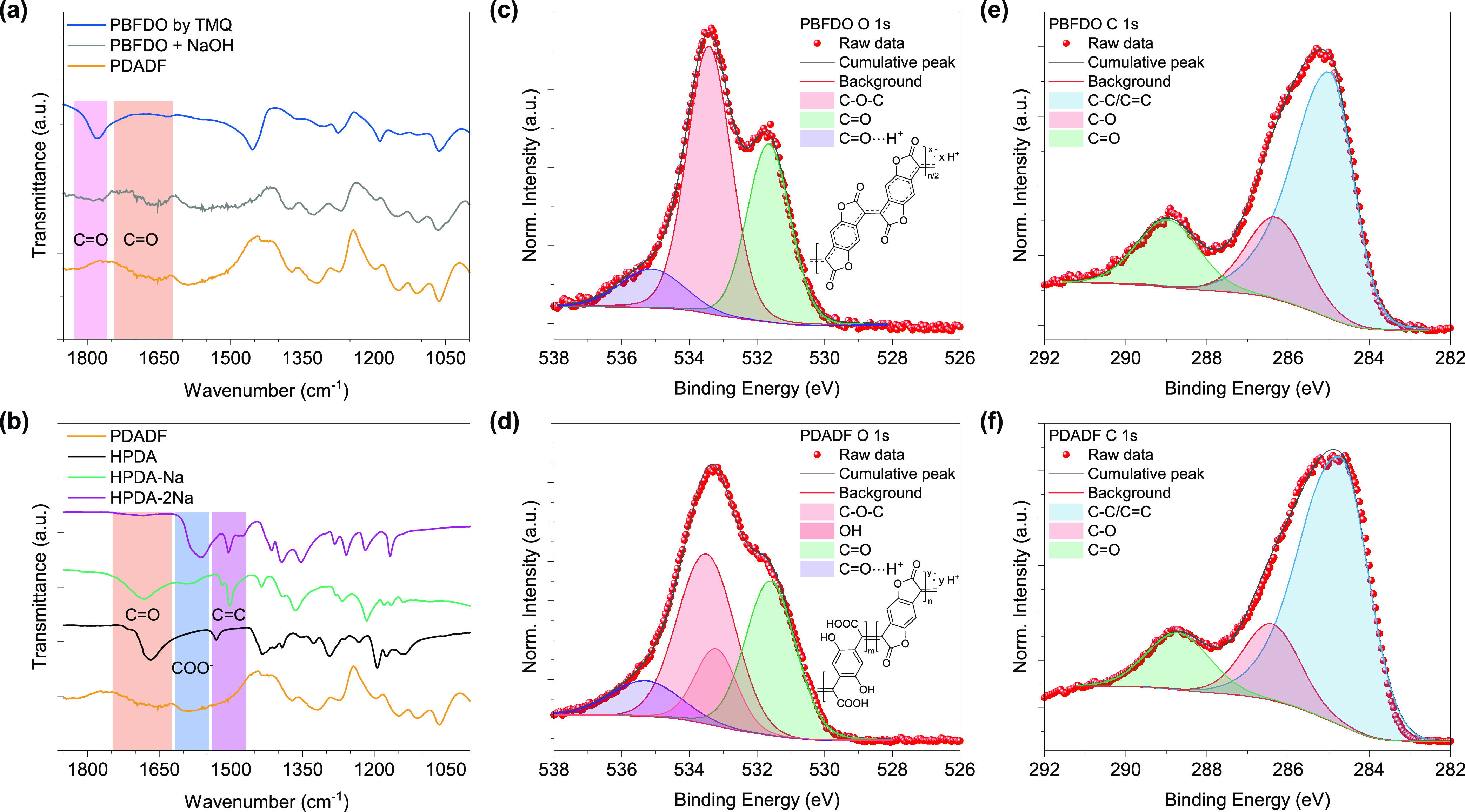
FTIR spectra of (a) PBFDO, PBFDO + NaOH (obtained from adding 1 eq. NaOH to an aqueous PBFDO dispersion), and PDADF and (b) a comparison of PDADF with HPDA and Na-substituted HPDA (both HPDA-Na and HPDA-2Na). (c–f) XPS spectra of PBFDO (c, e) and PDADF (d, f).

Next, we characterized the chemical structure of PDADF and its differences with PBFDO. The FTIR spectrum of PDADF exhibits several distinct features when compared to PBFDO (Figure 3a). While the latter displays a noticeable C=O stretching of the lactone moiety at 1781 cm^–1^, PDADF lacks a clear peak in the same region, as highlighted in light pink. Additionally, three broad features in the range of 1850–1776, 1752–1624, and 1623–1471 cm^–1^ are observed for PDADF. We attributed the features in the highlighted orange region at 1752–1624 cm^–1^ to the characteristic band of a carboxylic acid group.^51,52^ These features are qualitatively similar to those observed in the FTIR spectrum of the HBFDO monomer treated with 1 eq NaOH (Figure S5a), which is known to be unstable in aqueous alkaline environments and undergo ring opening,^53,54^ leading to the formation of sodium 2-(4-(carboxymethyl)-2,5-dihydroxyphenyl)acetate (HPDA-Na, Figure S6a). This observation is also consistent with the reduction of pH measured before (pH = 11.59) and after (pH = 4.98) polymerization. The addition of 1 eq NaOH to an aqueous dispersion of PBFDO polymerized by TMQ in DMSO (PBFDO + NaOH) reveals similar FTIR absorption features to those of PDADF, corroborating the existence of at least a partially open-ring structure in PDADF (Figure 3a).

To further understand the FTIR spectra of PDADF, we compared PDADF with ring-opened HBFDO (2,2′-(2,5-dihydroxy-1,4-phenylene)diacetic acid, HPDA) and its Na-substituted derivatives (HPDA-Na and HPDA-2Na, Figure 3b). ^1^H NMR analysis revealed minimal differences upon Na substitution of HPDA (Figure S7). The FTIR spectrum of HPDA-Na indicates the generation of a new asymmetric COO^–^ vibration [*v*_as_ (COO^–^)] at around 1621–1542 cm^–1^,^55^ which becomes more pronounced upon further carboxylic acid deprotonation for HPDA-2Na (Figure 3b, blue region). This *v*_as_ (COO^–^) peak, in conjunction with a broadened peak in the dark pink region (1541–1471 cm^–1^) corresponding to C=C stretching, underlies the features observed in PDADF (1621–1471 cm^–1^, blue and dark pink regions combined). The broader C=C stretching in PDADF, compared to HPDA derivatives, is to be expected from the formation of new C=C bonds between methylene groups during polymerization. The absorption of the C=C bond vibration in PBFDO is similarly broad (Figure 3a, ∼1450 cm^–1^). Furthermore, the characteristic band of the carboxylic acid group (orange, 1752–1624 cm^–1^) gradually shifts toward higher wavenumbers and lowers in intensity upon progressive Na substitution of HPDA (from HPDA to HPDA-Na to HPDA-2Na, Figure 3b), which could explain the broad features exhibited by PDADF in this region.

To gain further insights into PDADF’s structural differences in relation to PBFDO and to rationalize the absence of a clear lactone C=O peak in PDADF’s FTIR spectrum, we mixed PBFDO with different mass ratios of HPDA. A shift in the peak position of the C=O vibration at 1781 cm^–1^ was observed upon adding HPDA in larger quantities (Figure S5b). This corresponds to a strengthening of the C=O vibration which we hypothesize to occur when the H^+^ in doped PBFDO is in contact with a COO^–^(H^+^)/OH group on HPDA. The change is relatively minor due to the difficult incorporation of HPDA into the PBFDO, as indicated by the appearance of white crystals upon film drying. However, in the case of the partially ring-opened PDADF polymer, where lactone and acid groups are thoroughly mixed, the lactone C=O groups will be close by and at varying proximity to COO^–^(H^+^)/OH groups. This is expected to amount to a broadening and a more significant shift of the lactone C=O vibration for PDADF. Thus, based on the FTIR spectra of PBFDO mixed with HPDA (Figure S5b), we hypothesize that the lactone C=O vibration of PDADF has broadened and shifted to a higher wavenumber compared to PBFDO, resulting in the absorption observed at ∼1800 cm^–1^ (light pink region in Figure 3a). Note that when HPDA is mixed with PBFDO, the O–H vibration peak is lost or obscured in the gently sloped baseline (Figure S8a). Similarly, we find in PDADF that there is no clear O–H vibration present in the FTIR spectrum. We attribute this obscuring of the O–H vibration to broadening due to (disordered) H-bonding of the copolymer and/or to the formation of a quinone structure^56^ (see Figure S8 for more details).

X-ray photoelectron spectroscopy (XPS) was carried out for additional characterization of the PDADF chemical structure. The XPS O(1s) spectra of PDADF can be fitted with an additional peak at 533.2 eV (OH on phenol), in contrast to PBFDO (C=O at 531.7 eV, C–O–C at 533.4 eV, and C=O–H^+^ at 535.1 eV, see Figure 3c,d). This observation suggests that PDADF has a more complex chemical structure. Consequently, the C=O peak (green) in PDADF is broader and has a larger area compared to PBFDO, suggesting the combined presence of C=O from both ring-opened carboxylic acid, ring-closed lactone moieties, and possibly *p*-quinone in PDADF (Figure 3d). The C=O–H^+^ peak (purple), corresponding to the doped state of protons attached to the C=O groups,^43^ is visible in both PDADF and PBFDO polymers.

Regarding the C(1s) spectra, PDADF and PBFDO exhibit similar fitting peaks due to a comparable chemical environment of carbon, except for the different binding energies of the C–O peak. This observation aligns with the PDADF’s O(1s) spectra, exhibiting both C–OH and C–O–C peaks (Figure 3e,f). In summary, XPS suggests that PDADF might comprise both closed-ring and open-ring repeating units.

The ultraviolet–visible-near-infrared (UV–vis–NIR) absorption spectra of PBFDO, synthesized using both TMQ and TMQ-PA, exhibit a strong polar/bipolaron absorption that extends beyond 2000 nm.^43^ PDADF shows similar polar/bipolaron absorption but relatively stronger absorption below 600 nm, which we attributed to the ring-opened structure (Figure S9).

To further investigate the electronic properties of PDADF, near-edge X-ray absorption fine structure (NEXAFS) spectroscopy was employed to probe the transition from C and O core levels to the unoccupied states of PDADF. Figure S10 depicts the NEXAFS spectra of C and O K-edge of PDADF compared with those of PBFDO. It is evident that the spectral features in PDADF are highly consistent with those in PBFDO. Particularly noteworthy is the similarity in the O K-edge feature, which closely mirrors that of PBFDO (Figure S10a). These features are attributed to self-doping occurring in the O-related molecular structure in PBFDO, as previously reported.^43^ A slight difference is observed instead in C K-edge spectra (Figure S10b), where the first absorption in PBFDO corresponds only to the shoulder structure, indicating a change in the C-related molecular structure in PDADF, which we attributed to the ring-opened moiety. In addition, we observed a slight upshift in the absorption energy of PDADF compared to PBFDO. Assuming the same exciton binding energy in PDADF and PBFDO, this upshift suggests a slight decrease in the lowest unoccupied molecular orbital (LUMO) energy level of PDADF. The corresponding electron affinity (EA) is lower in PDADF than in PBFDO by about 0.1 eV. In general, the similar absorption features observed for PDADF and PBFDO indicate similar electronic properties in both polymers.

Building upon the analysis of the aforementioned FTIR, XPS, UV–vis–NIR, and NEXAFS data, we propose that the PDADF structure includes both ring-closed moieties, akin to PBFDO, and ring-opened moieties. In the latter, the lactone repeating unit opens, leading to the formation of carboxylic acid and phenol groups, facilitating polymer dispersion in water (see Figure S11). However, quantification of the ratio between these structures is challenging due to limitations in polymer characterization.

### Electrical Performance and Film Microstructure Characterization

**Figure 4:**
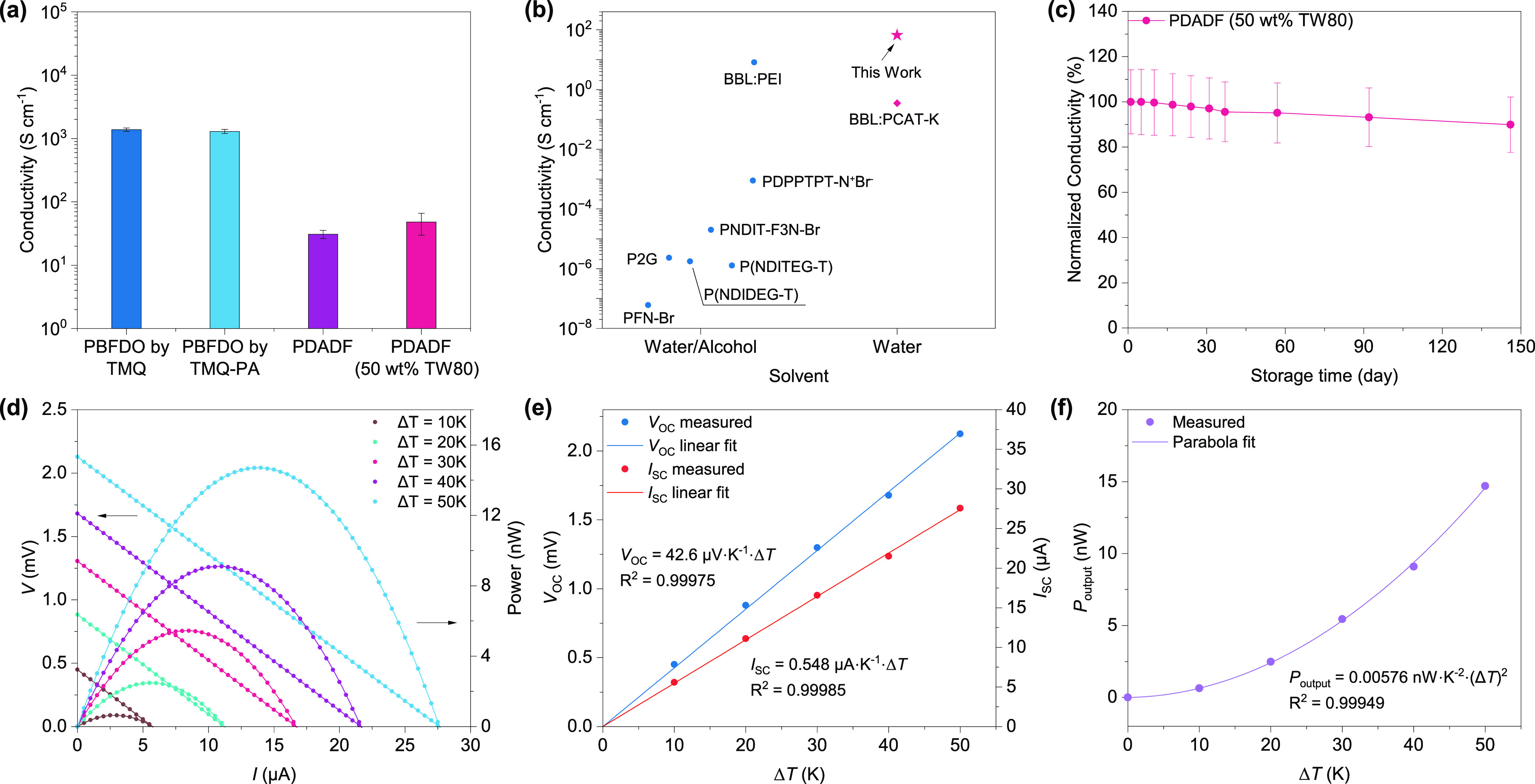
(a) Electrical conductivity of PBFDO and PDADF. (b) Conductivity comparison of *n*-type CPs based on the processing solvents. (c) Normalized PDADF (50 wt % TW80) thin film conductivity as a function of time stored at ambient. (d) Output voltage and power output of a TEG comprising PDADF as the *n*-type leg and PEDOT:PSS as the *p*-type leg at different Δ*T*. (e) Open-circuit voltage and short-circuit current of the TEG in (d). (f) Power output per *p*–*n* pair as a function of Δ*T*.

We then investigated the electrical and thermoelectric properties of PDADF and compared them to PBFDO (Figure 4a). The electrical conductivity of PBFDO films, processed from DMSO and measured by a four-point probe method, was determined to be approximately 1379 ± 83 S cm^–1^ for TMQ and 1297 ± 98 S cm^–1^ for TMQ-PA, respectively. This result confirms the catalytic efficiency of TMQ-PA to oxidize HBFDO in DMSO. In contrast, PDADF films drop-casted from water exhibited a lower electrical conductivity of 30.9 ± 4.6 S cm^–1^ and good reproducibility (Figure S12). We attributed the reduced electrical conductivity of PDADF with respect to PBFDO to the coarse morphology of the former when compared to the latter (see Figures S13–S15 and Table S2 for a full analysis of the microstructure of both polymers). The use of different amounts of NaOH during PDADF synthesis yields batches with varying electrical conductivity, with 1 eq affording a polymer with the highest conductivity in the series (Figure S16). The use of the surfactant Tween 80 (TW80) yields more homogeneous films (Figure S13), reaching electrical conductivities as high as 66 S cm^–1^ (average 48 ± 18 S cm^–1^, see Table 1 and Figure S17 for a survey of different surfactants). Despite these values being lower than those measured for PBFDO in DMSO, they are the highest reported for *n*-type conducting polymers synthesized and processed from water or water/alcohol mixtures^33−36,38,41^ (Figure 4b) and among the highest for *n*-type conducting polymers entirely processed in ambient conditions. Remarkably, the electrical conductivity of PDADF synthesized using recycled TMQ-PA is on par with that of PDADF produced using freshly synthesized oxidant (Table 1, entry 4), highlighting the robustness and effectiveness of the recycling process.

**Table 1:** Summary of the Electrical Conductivity Values

entry	polymer	catalyst	solvent	base	surfactant	conductivity (S cm^–1^)
1	PBFDO	TMQ	DMSO			1379 ± 83
2	PBFDO	TMQ-PA	DMSO			1297 ± 98
3	PDADF	TMQ-PA	H_2_O	NaOH		30.9 ± 4.6
4^a^	PDADF	TMQ-PA	H_2_O	NaOH		30.8 ± 4.8
5	PDADF	TMQ-PA	H_2_O	NaOH	TW80 (50 wt %)	48 ± 18

a PDADF synthesized from recycled TMQ-PA.

The Seebeck coefficient of PDADF was found to be −12.25 ± 1.16 μV K^–1^ (Figure S18). This value slightly reduces to −10.52 ± 1.02 μV K^–1^ when TW80 is used as the surfactant. For comparison, the Seebeck coefficient of PBFDO films was found to be around −20 μV K^–1^, in agreement with previous reports.^43^ The negative sign of the Seebeck coefficient values is consistent with electrons being the majority charge carriers.

Typically, *n*-type conducting polymers are susceptible to degradation when processed and operated in air. This degradation is primarily attributed to the quenching of radical anions by atmospheric moisture and oxygen.^57^ The air stability of PDADF films was studied by monitoring variations in electrical conductivity over time and under ambient conditions. Thin films of PDADF (71 ± 9 nm thick, 50 wt % TW80) exhibited an impressive 90% retention of the electrical conductivity after 146 days (Figure 4c). This result is particularly noteworthy for *n*-type conjugated polymers synthesized and processed entirely from water. Additionally, PDADF demonstrates remarkable thermal stability (Figure S19), no observable phase transitions up to 250 °C (Figure S20), and good electrochemical properties (Figures S21 and S22).

### Applications

Next, we evaluated the possibility of fabricating the first fully water-processable, flexible organic thermoelectric generators (OTEGs). An aqueous dispersion of PDADF (50 wt % TW80) was used to fabricate the *n*-leg, while PEDOT:PSS (5 vol % EG) was used as the *p*-leg. A polyethylene naphthalate (PEN) foil (100 μm thick) was used as the flexible substrate, with gold electrodes patterned on it via evaporation through a shadow mask (see Experimental Section for further details). Subsequently, PDADF and PEDOT:PSS were drop-casted through a mask. Importantly, all processes and measurements were conducted in air without encapsulation. These flexible OTEGs processed from water exhibit an internal resistance of 78 ohms (Figure S23) and demonstrate open-circuit voltage and short-circuit current responses linearly proportional to the applied temperature gradient (Δ*T*), with a remarkable thermovoltage of 42.6 μV K^–1^ (Figure 4e). The power output per *p*–*n* pair of the TEG exhibits a quadratic relationship with the temperature gradient, ranging from 0.64 nW (Δ*T* = 10 K) to 14.7 nW (Δ*T* = 50 K, see Figure 4f and Table S3).

## Conclusions

In conclusion, we reported the synthesis of the conducting copolymer PDADF by employing a water-soluble organic catalyst, TMQ-PANa. The utilization of this catalyst not only enables the polymerization of HBFDO in pure water but also allows for the efficient recovery of the catalyst postreaction. This stands in contrast to previous methods where catalyst consumption during the polymerization process hindered recovery. PDADF exhibits remarkable electrical conductivity, surpassing 48 ± 18 S cm^–1^ (with a maximum of 66 S cm^–1^), positioning it among the highest reported for *n*-type polymers that are both synthesized in and processed from water. Moreover, the spin-cast film demonstrates outstanding air stability, retaining 90% of the initial conductivity after 146 days in ambient without encapsulation. To underscore the potential of PDADF in the development of efficient and stable electronic devices, we incorporated PDADF as the *n*-leg material in fully water-processable OTEGs, achieving power outputs of approximately 15 nW at Δ*T* = 50 K. This work not only enhances our understanding of water-based synthesis methods but also paves the way for the sustainable development of high-performance electronic materials and devices.

## Supporting Information Available

Synthetic procedures and characterization data of PDADF; details of the device fabrication; and NMR spectra, FTIR spectra, UV–vis–NIR spectra, TGA, DSC, NEXAFS, GIWAXS, and AFM images (PDF)
