# Supplementary material for: A Highly Conductive n-Type Conjugated Polymer Synthesized in Water
Source: J Am Chem Soc. 2024 May 30;146(23):15860–8. doi: 10.1021/jacs.4c02270 (PMC11177263; doi:10.1021/jacs.4c02270)
Supplement: Supplementary file 1 — ja4c02270_si_001.pdf [file ja4c02270_si_001.pdf]

# Supporting Information

## A highly conductive n-type conjugated polymer synthesized in water

Qifan Li,[1,‡] Jun-Da Huang,[1,2,‡] Tiefeng Liu,[1,3] Tom P. A. van der Pol,[1] Qilun Zhang,[1,2] Sang Young Jeong,[4] Marc-Antoine Stoeckel,[1,3,5] Han-Yan Wu,[1] Silan Zhang,[1,2] Xianjie Liu,[1] Han Young Woo,[4] Mats Fahlman,[1,2] Chi-Yuan Yang[1,5,]* and Simone Fabiano[1,2,3,5,]*

[1]Laboratory of Organic Electronics, Department of Science and Technology, Linköping University, SE-60174 Norrköping, Sweden. E-mail: chi-yuan.yang@liu.se, simone.fabiano@liu.se

[2]Wallenberg Wood Science Center, Department of Science and Technology, Linköping University, SE-60174, Norrköping, Sweden.

[3]Wallenberg Initiative Materials Science for Sustainability, Department of Science and Technology, Linköping University, SE-60174, Norrköping, Sweden.

[4]Department of Chemistry, College of Science, Korea University, Seoul 136-713, Republic of Korea.

[5]n-Ink AB, Bredgatan 33, SE-60221 Norrköping, Sweden

[‡]Q. L. and J.-D. H. contributed equally to this work.

Experimental Section ..... 2
Synthesis ..... 2
Film casting ..... 2
Absorption spectra ..... 2
XPS and NEXAFS spectroscopy ..... 3
Grazing-incidence wide-angle X-ray scattering and AFM characterization ..... 3
Thermoelectric generators ..... 3
Electrical characterization ..... 3
Organic electrochemical transistors (OECTs) and inverters ..... 4
General materials characterization ..... 4
Supplementary Figures and Tables ..... 5
References ..... 32

# Experimental Section

## Synthesis

All chemical reagents were purchased from Sigma-Aldrich and used as received unless otherwise indicated. Duroquinone (TMQ) was purchased from TCI EUROPE N.V. PEDOT:PSS (Clevios PH1000) was purchased from Heraeus Holding GmbH.

3-(2,4,5-trimethyl-3,6-dioxocyclohexa-1,4-dien-1-yl)propanoic acid (TMQ-PA) was synthesized according to previous literature.[1] 3,7-dihydrobenzo[1,2-b:4,5-b']difuran-2,6-dione (HBFDO) was synthesized as previously reported.[2]

Poly[(2,2'-(2,5-dihydroxy-1,4-phenylene)diacetic acid)-co-3,7-dihydrobenzo[1,2-b:4,5-b']difuran -2,6-dione] (PDADF) synthesis: TMQ-PA (7.89 mmol, 1.75 g, 1 eq) was added to a 250 mL round-bottom flask with a stir bar, following by freshly made 0.5 M NaOH (7.89 mmol, 15.8 mL, 1 eq), and stirred at room temperature (RT) for 10 min until all TMQ-PA dissolved. Another 34.2 mL DI water was added to the solution, forming 30 mg/mL dispersion according to monomer HBFDO. HBFDO (7.89 mmol, 1.50 g, 1 eq) was added to the diluted solution and stirred at 100 °C for 3.5 h. The final suspension was diluted with water after cooling down to RT and extracted with diethyl ether 5 times until the organic phase was colorless. All the diethyl ether was collected and purified by column chromatography to get the recycled mixture of catalyst and its precursor, followed by the oxidation to yield TMQ-PA. The PDADF aqueous phase was collected by centrifugation (6000 rpm for 10 min) and washed with DI water for other 5 times. PDADF was collected from the bottom of the centrifuge tube, forming about 20-25 mg/mL water dispersion (the yield is around 20-25%).

PDADF (50 wt% TW80) synthesis: PDADF was synthesized based on the same aforementioned method, followed by the addition of 0.75 g TW80 (0.5-time mass multiple according to HBFDO). The mixture was stirred for another 2 days to form the final PDADF (50 wt% TW80) water ink.

Poly(benzodifurandione) (PBFDO) was synthesized using TMQ or TMQ-PA catalysts, following a procedure reported before.[3]

## Film casting

PDADF/PDADF (50 wt% TW80) water dispersion was drop-cast on prewashed and plasma-cleaned substrates like glass, Au, Si, Si/$SiO_2$. PDADF (50 wt% TW80) was spin-cast (1500 rpm, 2 mins, acceleration 1500 rpm $s^{-1}$). All these films were submerged into acetone overnight to remove impurities and dried with a nitrogen gun.

PBFDO film (by TMQ or TMQ-PA) was spin-cast (1500 rpm, 2 mins, acceleration 1500 rpm $s^{-1}$, then 3000 rpm, 10 s, acceleration 3000 rpm $s^{-1}$), and the films were immediately barked on a 40 °C hot plate for 20 mins.

## Absorption spectra

All the Fourier-transformed infrared (FTIR) spectra samples were prepared by evaporating solvent to form a solid sample on a 70 °C hotplate and measured with PerkinElmer Spectron 3 in ATR

mode. The ultraviolet-visible-near-infrared (UV-vis-NIR) absorption spectroscopy measurements were performed using a Perkin Elmer Lambda.

## XPS and NEXAFS spectroscopy

The samples were deposited in ambient on Au substrates by drop-casting, followed by washing with acetone and water, and then quickly transferred into the load lock chamber of the ultrahigh vacuum (UHV) system for the following steps. X-ray photoemission spectroscopy (XPS) was performed with a Scienta-200 hemispherical analyzer using a monochromatized Al Kα source with a photon energy of 1486.6 eV. All photoelectron spectroscopy measurements were carried out with a base pressure lower than $1 \times 10^{-9}$ mbar. Near edge x-ray absorption fine structure (NEXAFS) spectroscopy measurement was performed at AU-Matline in ASTRID2 synchrotron source, Aarhus University, Denmark, in partial electron yield mode recording C, O Auger electrons using hemispherical analyzer operated in angular mode with a pass energy of 100 eV.

## Grazing-incidence wide-angle X-ray scattering and AFM characterization

GIWAXS experiments were performed following the previous procedure.[4] The samples were measured at Beamline 9A at the Pohang Accelerator Laboratory in South Korea. The X-ray energy was 11.08 eV, and the incidence angle was 0.12°. Samples were measured in vacuum, and the total exposure time was 10 s. The scattered X-rays were recorded by a charge-coupled device detector located 220.1727 mm from the sample. AFM images were recorded with an Icon XR from Bruker, using a silicon nitride cantilever with a spring constant of 40 N $m^{-1}$.

## Thermoelectric generators

The TEGs had one p/n-leg pair module with the in-plane geometry design on the 100 μm-thick polyethylene naphthalate (PEN) substrate. PEDOT:PSS (PH1000) treated with ethylene glycol (EG 5 vol%) was used for the p-leg. The width of the p/n legs was set to 2 mm/10 mm due to the different conductivity of p/n materials; the leg length and thickness were both 2.5 mm and 1.5 μm, respectively. First, Cr/Au (5 nm/ 50 nm) were deposited onto the PEN substrate by evaporation through a shadow mask. Then, PDADF (50 wt% TW80) was cast on PEN in air, followed by submersion in acetone to remove impurities, and dried with a nitrogen gun. Next, PEDOT:PSS with EG was printed by drop casting. All the processes and measurements were done in air without encapsulation.

## Electrical characterization

Electrical conductivity was measured by a four-point probe technique. The conductivity was calculated through the following equation: $\sigma = IL/VWd$, where $W$ is the sample width, $d$ is the thickness of the film, and $L$ is the length between two electrodes, $I$ is the applied current and $V$ is the measured voltage. Here, $W = 7$ mm, $L = 3$ mm, and $d$ is dependent on different samples. The Seebeck coefficients were measured inside the glovebox by a pair of Peltier elements to provide a temperature difference. All these measurements used a Keithley 4200-SCS semiconductor characterization system.

## Organic electrochemical transistors (OECTs) and inverters

OECTs were fabricated following a procedure reported previously.[4] The devices have a width and length of 100 μm and 12 μm, respectively. The inverters were assembled by connecting a 4 kΩ resistor in series with a PDADF-based OECT. The OECTs and inverters were characterized using a Keithley 4200A-SCS, utilizing 0.1 M NaCl electrolyte and Ag/AgCl pellet gate electrodes.

## General materials characterization

The Nuclear magnetic resonance (NMR) data were collected from a 500 MHz Bruker system. The thermogravimetric analysis (TGA) was performed by TA Q500. The samples were heated from 50 °C to 850 °C with a heating rate of 10 °C $min^{-1}$ under a nitrogen atmosphere. The differential scanning calorimetry (DSC) was performed by TA DSC250 under nitrogen flow at heating/cooling rates of 10/10 °C $min^{-1}$. The zeta potential of the solutions was characterized by dynamic light scattering at Zetasizer Nano ZS90 (laser wavelength = 632.8 nm) at room temperature. All solutions are diluted to about 0.1 mg $ml^{-1}$ (without filtering) and sonicated for 5 min before testing. Cyclic voltammetry (CV) was performed using a Potentiostat BioLogic SP-200. CV measurements were performed using 0.2 M tetrabutylammonium bis-trifluoromethane-sulfonimidate (TBA-TFSI) in propylene carbonate.

## Supplementary Figures and Tables

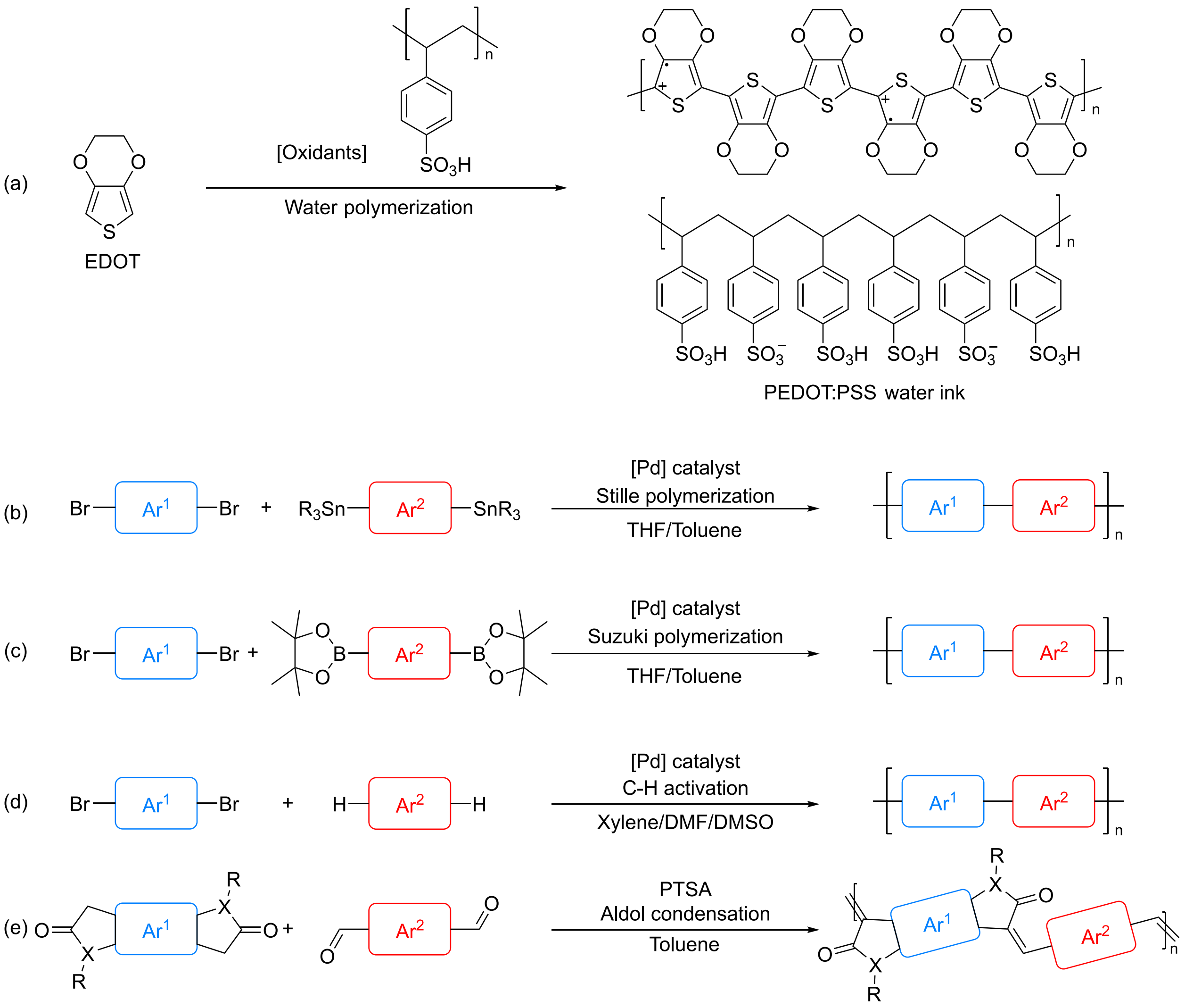


**Figure S1.** Typical synthesis methods to prepare p-type and n-type CPs (a) PEDOT synthesis from water in the presence of PSS to form PEDOT:PSS water ink. (b) Stille polymerization of n-type CPs, Pd catalysts, and problematic solvents such as THF and toluene are typically used. (c) Suzuki polymerization of n-type CPs. (d) C-H activation of n-type CPs. (e) Aldol condensation of n-type CPs.

**Table S1.** Summary of representative n-type conjugated polymers polymerized or processed from water/alcohol-based solvents.

| Materials | Solvent for polymerization | Solvent for processing | Conductivity (S/cm) | Year | Ref. |
|---|---|---|---|---|---|
| PDADF | water | water | 30.9 ± 4.6 | 2024 | This work |
| PDADF (50 wt% TW80) | water | water | 48 ± 18 | 2024 | This work |
| BBL:PCAT-K | PPA | water | 0.35 | 2023 | 5 |
| BBL:PEI | PPA | EtOH | 8 | 2021 | 6 |
| PFN-Br | toluene/THF | EtOH/MeOH | N.A. | 2021 | 7 |
| P(NDIDEG-T) | toluene | water:EtOH | $1.75\times10^{-6}$ | 2021 | 8 |
| P(NDITEG-T) | toluene | water:EtOH | $1.28\times10^{-6}$ | 2021 | 8 |
| P2G | chlorobenzene | EtOH | $2.3\times10^{-6}$ | 2021 | 9 |
| PDPPTBT | toluene:water | water | N.A. | 2020 | 10 |
| PDPPTPT-$N^{+}Br^{-}$ | THF | water/MeOH | $8.87\times10^{-4}$ | 2018 | 11 |
| PNDIT-F3N–F | toluene/THF/DMF | EtOH/MeOH | N.A. | 2017 | 12 |
| PNDIT-F3N-Br | toluene/THF/DMF | EtOH/MeOH | N.A. | 2016 | 13 |

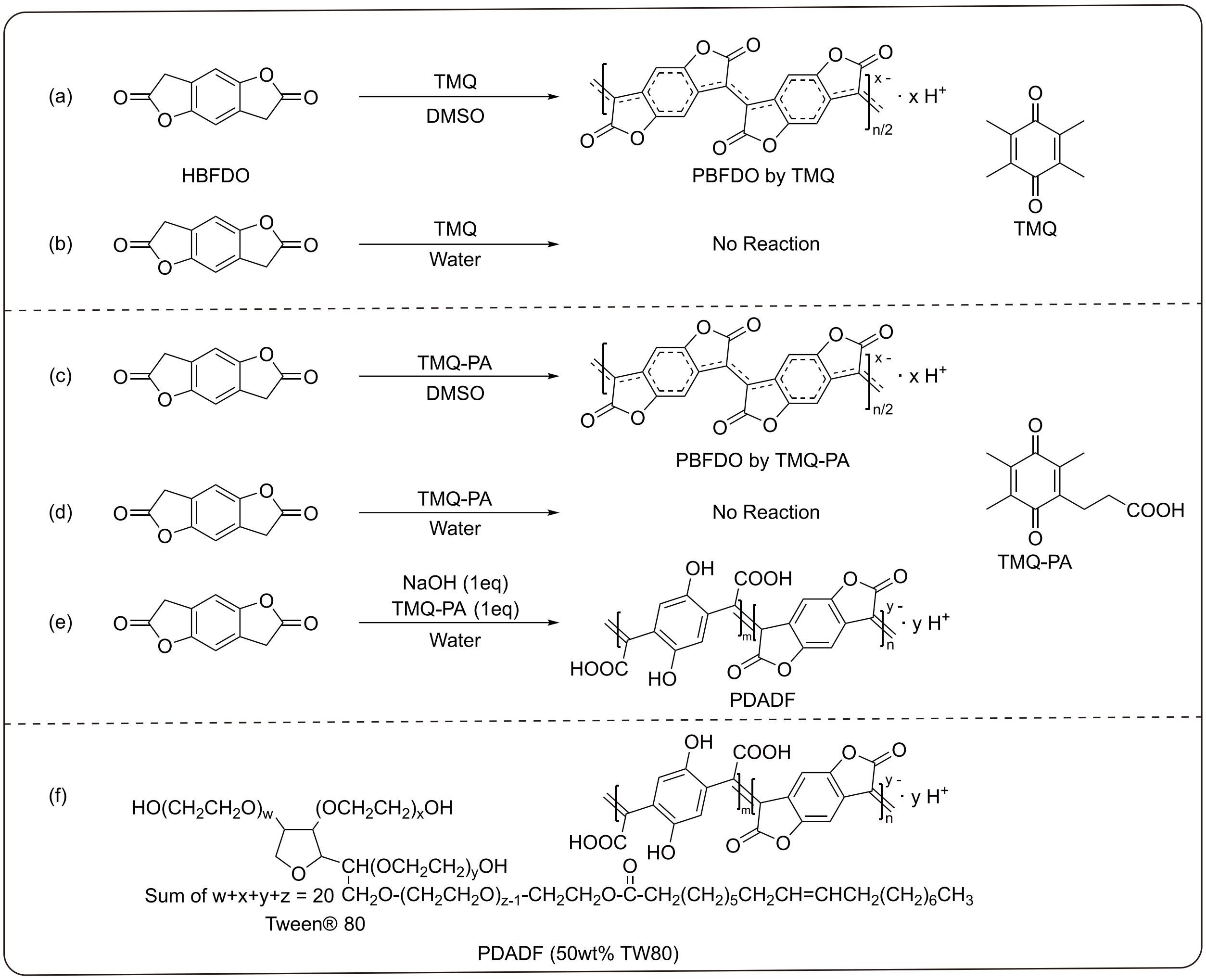


**Figure S2.** (a) Synthesis of PBFDO using TMQ as the catalyst, following Tang's protocol.[3] (b) Attempting to replace DMSO with water in the aforementioned synthesis resulted in the absence of polymerization when TMQ was utilized as the catalyst. (c) PBFDO synthesized from DMSO using TMQ-PA, highlighting the catalytic capability of TMQ-PA for both polymerization and in-situ doping. (d) No polymerization was observed in water, employing TMQ-PA as the catalyst. (e) PDADF was successfully polymerized in water with TMQ-PANa as the catalyst. (f) Use of Tween 80 (TW80) surfactant to stabilize the water-based PDADF ink (50 wt% TW80).

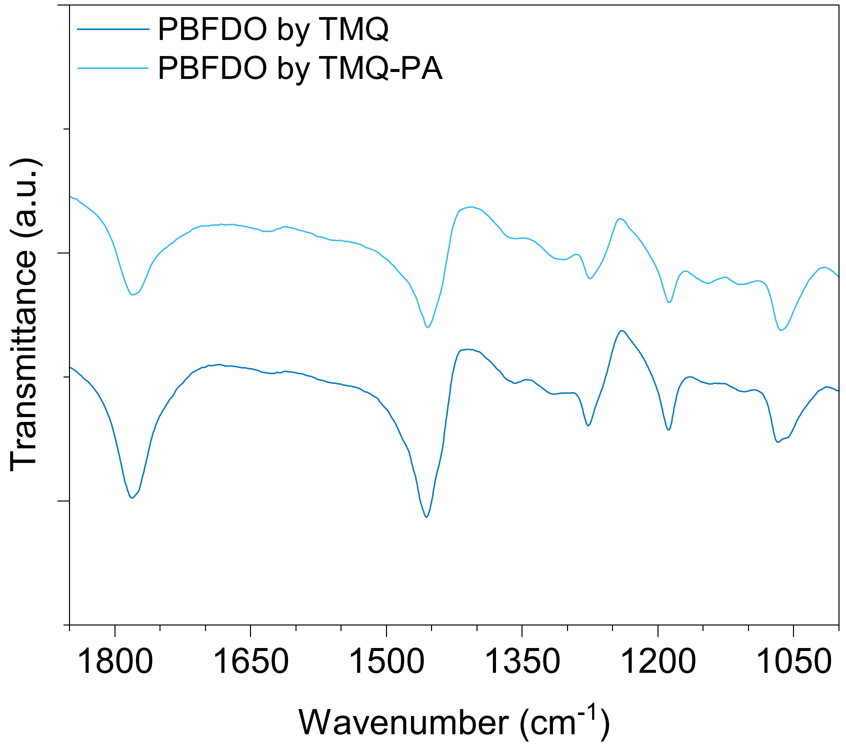


**Figure S3.** Identification of PBFDO synthesized from TMQ and TMQ-PA by offset FTIR spectra of PBFDO synthesized from TMQ and TMQ-PA, indicating that PBFDO was formed regardless of catalyst.

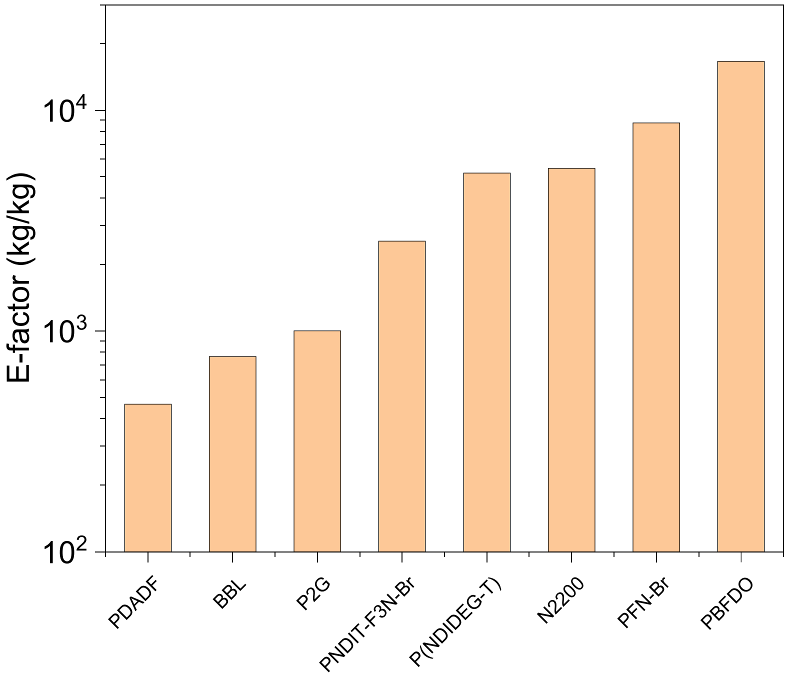


**Figure S4.** E-factor for the synthesis of PDADF and comparison with PBFDO and other prominent n-type polymers (note: only polymerization and purification steps were considered here).[3,6,7,9,13,14]

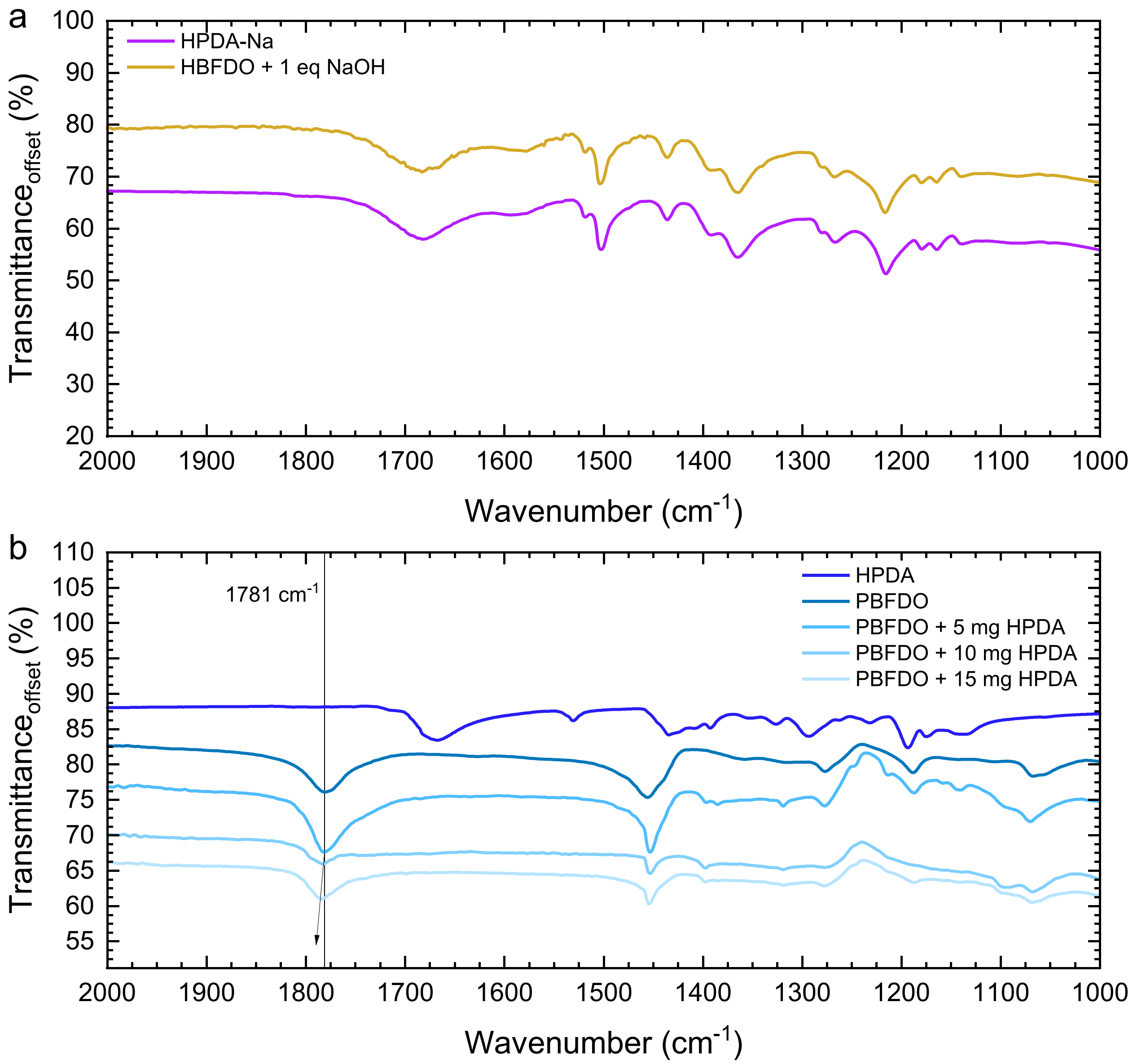


**Figure S5.** FTIR spectra. (a) Adding 1 eq NaOH to HBFDO water dispersion formed a fully ring-opened structure HPDA-Na, which reveals that the HBFDO was unstable in the base solution. (b) Different mass ratios of HPDA were added to 5 mg/mL PBFDO DMSO ink and stirred overnight. After the films had dried, some small white crystals could be observed in the film. These spectra are recorded with ATR-FTIR. Hence, differences in signal strength can be attributed to variations in contact between the polymer powder and the diamond crystal, likely resulting from differences in coverage or applied pressure.

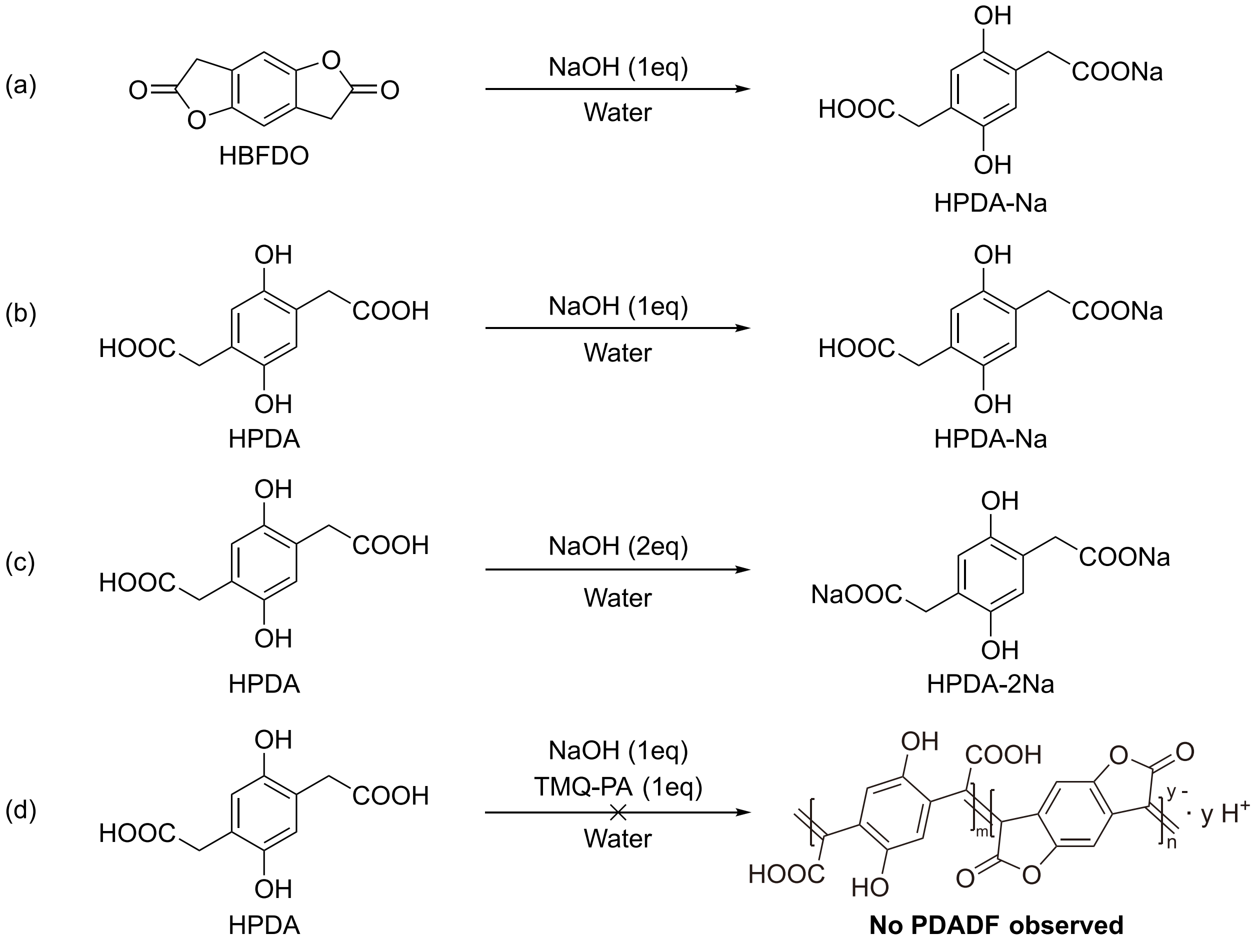


**Figure S6.** (a) HBFDO is unstable in a basic environment, and a new compound, HPDA-Na, is formed. (b) Adding 1 eq NaOH induces the formation of HPDA-Na. (c) Adding 2 eq NaOH induces the formation of HPDA-2Na. (d) No PDADF was observed when HPDA was used as the monomer instead of HBFDO under the standard reaction conditions for synthesizing PDADF.

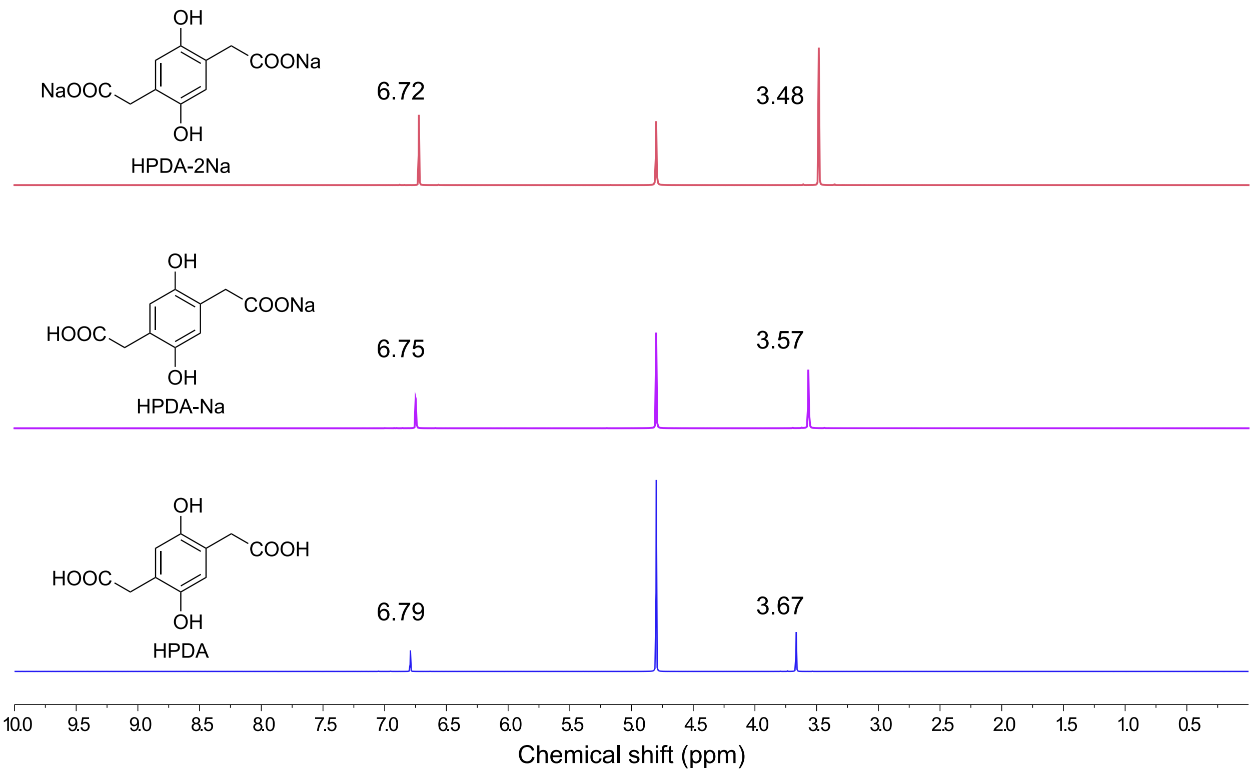


**Figure S7.** $^{1}H$ NMR spectra of HPDA, HPDA-Na, and HPDA-2Na. $^{1}H$ NMR spectra indicate that the formation of sodium salt causes a slight shift of the methylene group.

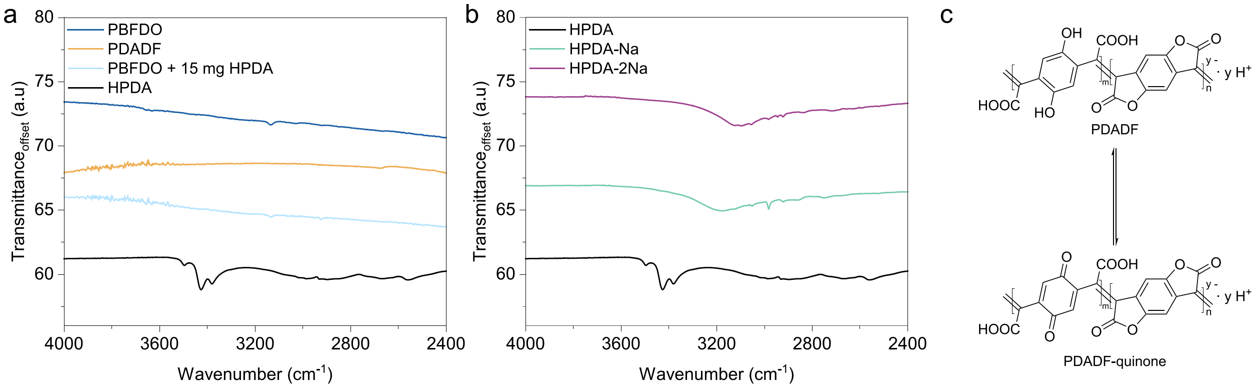


**Figure S8**. (a) Comparison of PBFDO and PDADF's FTIR spectra in the OH vibration region. (b) FTIR spectra of HPDA derivatives relative to the OH vibration region. (c) PDADF in equilibrium with its quinone structure. We observe that HPDA shows appreciable O-H vibrations spanning 3300-3500 cm$^{-1}$. However, when HPDA is mixed with PBFDO, we note that the O-H vibration is lost or obscured in the gently sloped baseline (a). Similarly, we find in PDADF that there is no clear O-H vibration present in the FTIR spectrum. We attribute this obscuring of the O-H vibration to broadening due to (disordered) H-bonding of the copolymer and/or to the formation of a quinone structure. In both cases, the O-H vibration is expected to broaden to an extent where it is difficult to discern. We find a similar trend for HPDA, HPDA-Na, and HPDA-2Na, whereby further Na substitution causes an increased shift and broadening of the O-H vibration (b). This could be similarly ascribed to increased H-bonding of the molecules HPDA-Na and HPDA-2Na. The formation of a PDADF quinone structure (c) could not be ruled out, which might be another explanation for the obscuring/broadening O-H vibration.

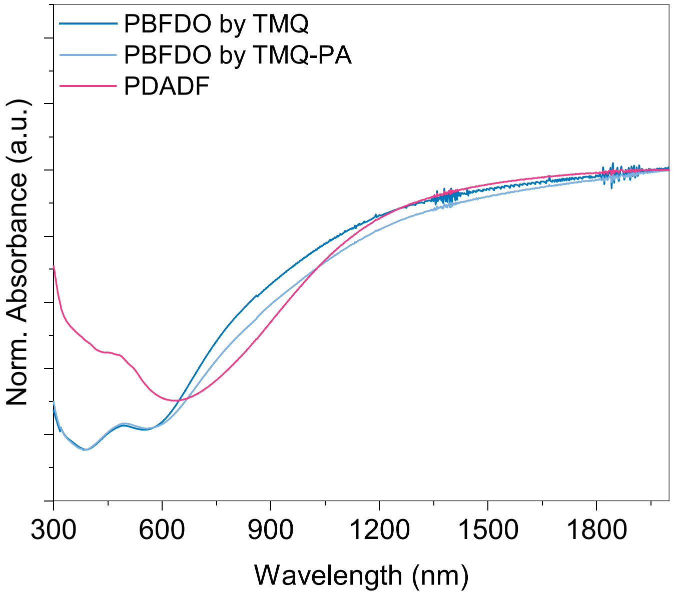


**Figure S9.** UV-vis-NIR absorption spectra of PDADF and PBFDO.

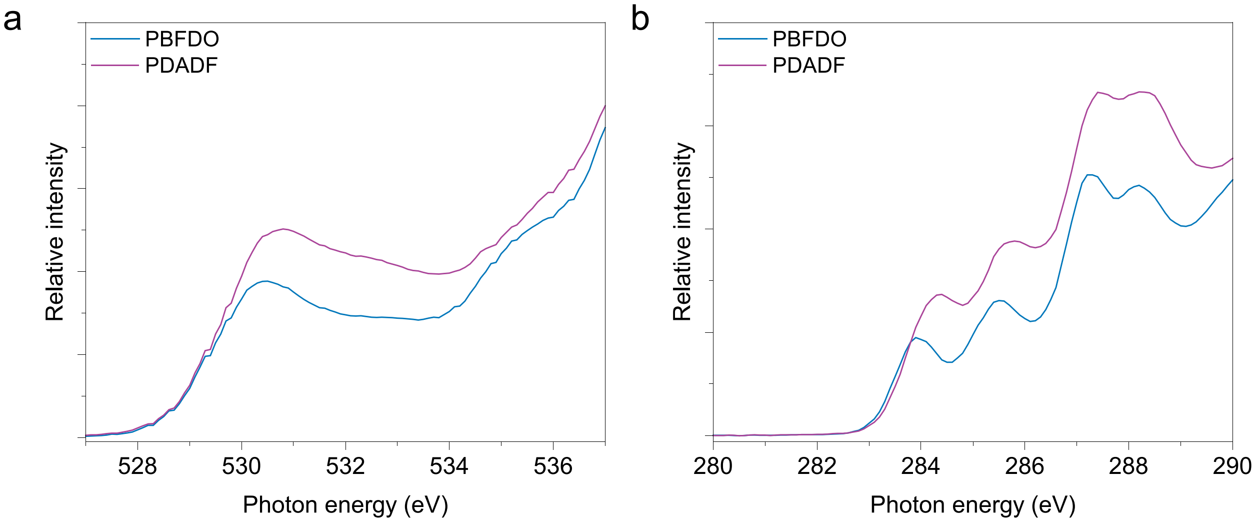


**Figure S10.** X-ray absorption spectroscopy (XAS) was employed to probe the transition from C and O core levels to the unoccupied states of PDADF and PBFDO. The similar C and O K-edges of PDADF compared with those of PBFDO reveal that they have very closed electronic properties. The slight differences in C K-edge spectra (b) on the first shoulder suggest changes in molecular structure of PDADF compared to PBFDO.

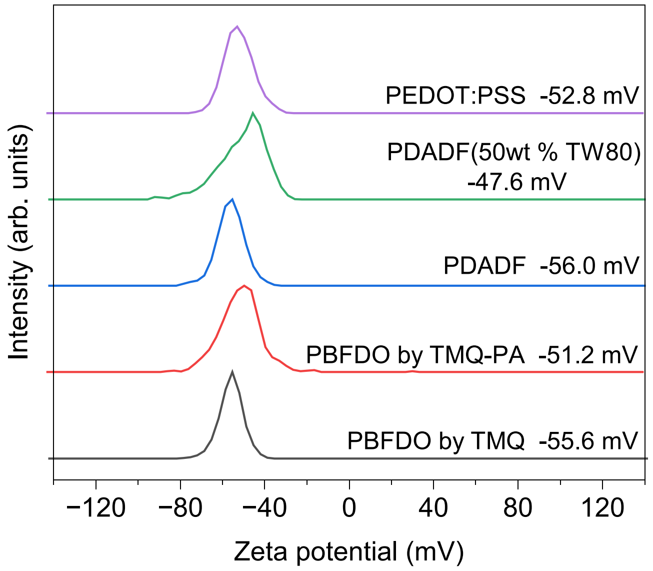


**Figure S11.** Zeta potential of PEDOT:PSS, PBFDO, PDADF, and PDADF (50 wt% TW80).

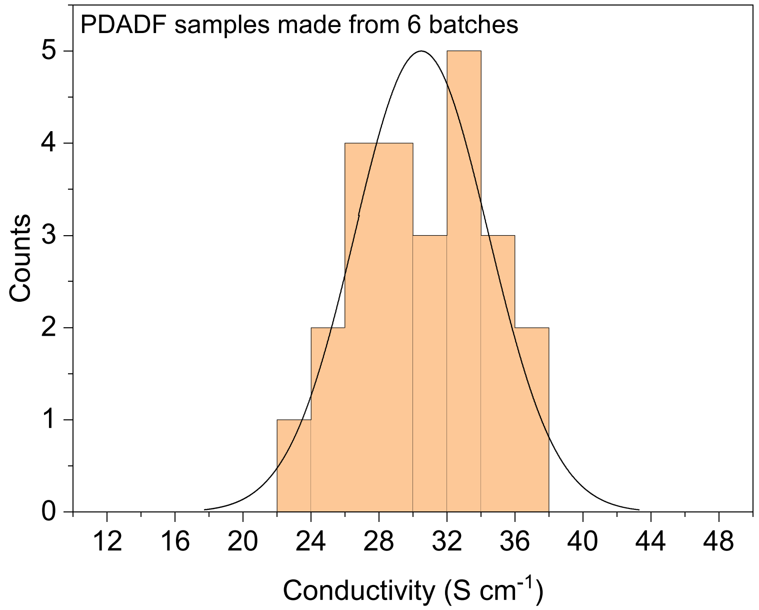


**Figure S12.** Batch-to-batch electrical conductivity variations based on 24 samples made from 6 batches of PDADF.

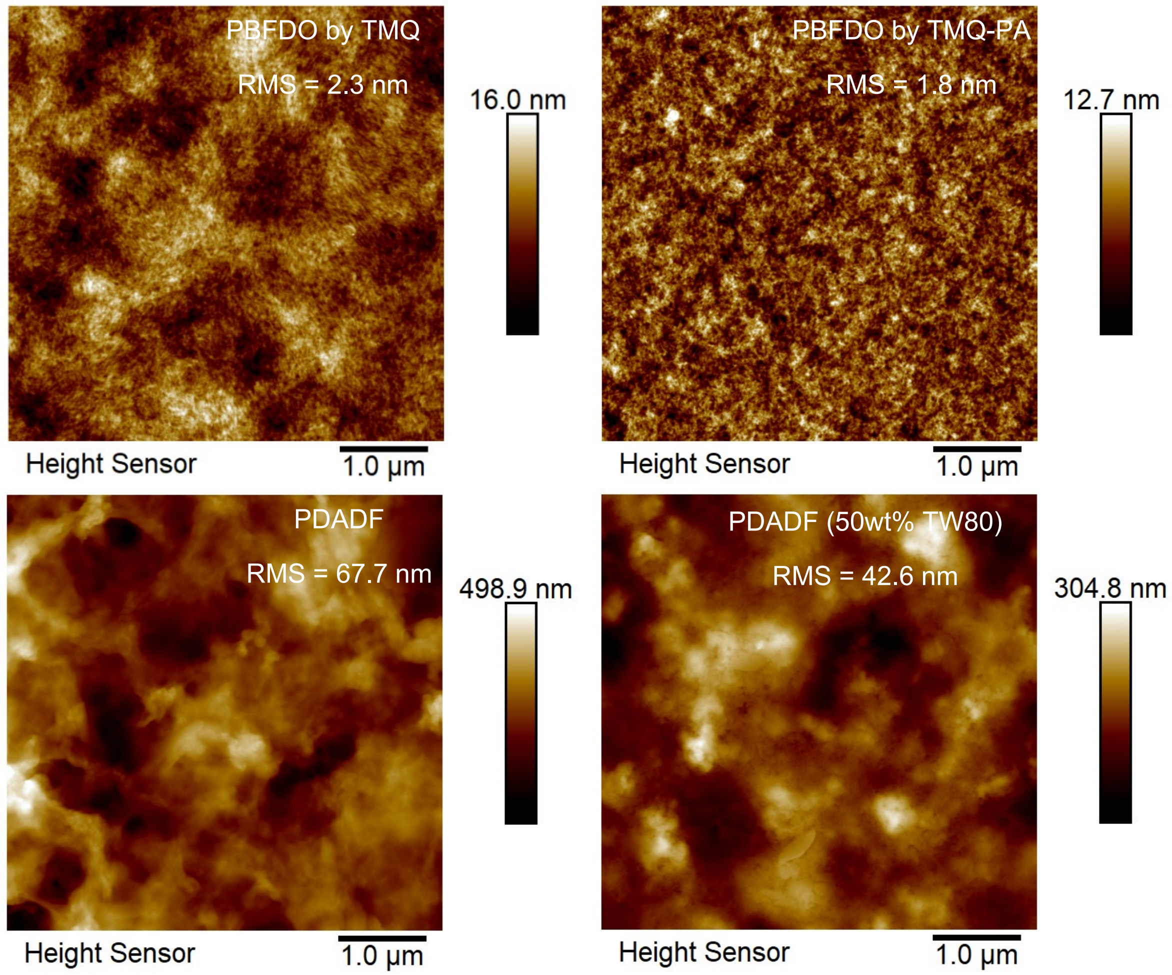


**Figure S13.** (*Top panels*) AFM height images of PBFDO synthesized using TMQ and TMQ-PA; (*Bottom panels*) AFM height images of PDADF and PDADF (50 wt% TW80) synthesized in water. PBFDO shows smooth and uniform film morphology, with a roughness of around 2 nm and negligible particles on its surface compared to PDADF. On the contrary, PDADF exhibits a much rougher surface (~70 nm) with aggregates. It is important to note that mixing PDADF with TW80 lowers its film roughness by more than a third (42.6 nm) and slightly increases film uniformity.

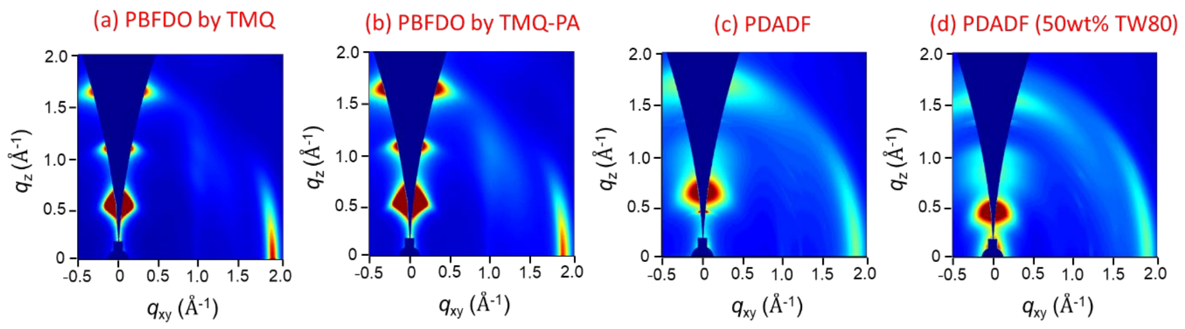


**Figure S14.** 2D GIWAXS patterns of PBFDO (a, b) and PDADF (c, d). The GIWAXS data reveal that PBFDO and PDADF films have similar edge-on orientation with respect to the substrate, with PDADF showing a weaker π-π stacking (010) signal compared to PBFDO (see Fig. S15a). PDADF exhibits a lamellar packing at $q_z$ = 0.657 Å$^{-1}$ ($d$-spacing = 9.53 Å), increasing to a $d$-spacing of 13.46 Å upon the addition of TW80 (Fig. S15b). Additionally, both PDADF and PDADF:TW80 (50 wt%) show reduced π-π stacking crystallinity with a decreased coherence length and increased paracrystalline disorder, both in-plane and out-of-plane, compared to PBFDO (see Table S2).

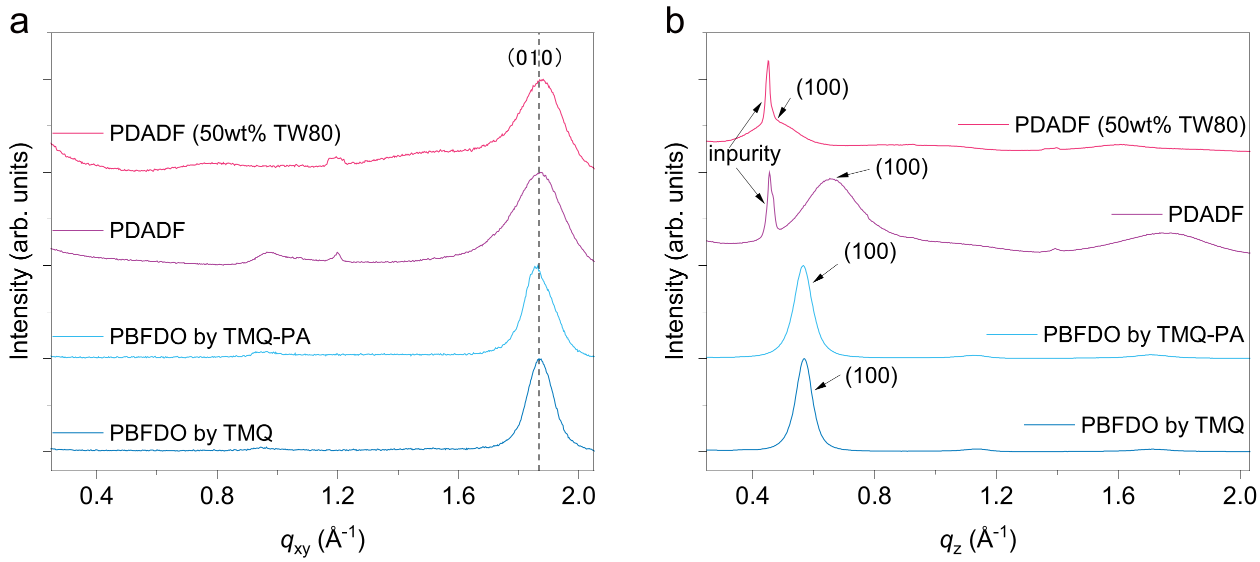


**Figure S15.** (a) In-plane and (b) out-of-plane GIWAXS line cuts of PBFDO (synthesized by TMQ or TMQ-PA), PDADF, and PDADF (50 wt% TW80) films.

**Table S2.** Summary of the calculated π-π stacking and lamellar distances and FWHM of (010) and (100) peaks from GIWAXS 1D data.

| Polymers | π-π stacking | | | | Lamellar stacking | | | |
|---|---|---|---|---|---|---|---|---|
| | $q_{xy}$ (010) (Å$^{-1}$) | $q_{xy}$ (010) (Å) | Coherence length (Å) | Paracrystalline disorder (Å) | $q_z$ (100) (Å$^{-1}$) | $q_z$ (100) (Å) | Coherence length (Å) | Paracrystalline disorder (Å) |
| PBFDO by TMQ | 1.871 | 3.358 | 24.63 | 0.1390 | 0.5688 | 11.046 | 42.492 | 0.1919 |
| PBFDO by TMQ-PA | 1.862 | 3.375 | 19.11 | 0.1582 | 0.5657 | 11.107 | 37.73 | 0.2042 |
| PDADF | 1.867 | 3.363 | 11.86 | 0.2004 | 0.6589 | 9.536 | 11.92 | 0.3366 |
| PDADF (50 wt% TW80) | 1.874 | 3.352 | 13.03 | 0.1909 | 0.4666 | 13.465 | 13.26 | 0.3793 |

a

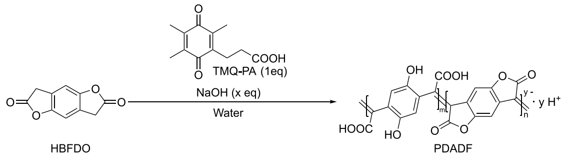


| Entry | HBFDO (eq) | TMQ-PA (eq) | NaOH (eq) | Conductivity (S cm$^{-1}$) |
|---|---|---|---|---|
| 1 | 1.0 | 1.0 | 0.2 | 0.053± 0.024 |
| 2 | 1.0 | 1.0 | 0.5 | 9.3± 0.49 |
| 3 | 1.0 | 1.0 | 1.0 | 35± 1.3 |
| 4 | 1.0 | 1.0 | 1.5 | 0.67± 0.11 |
| 5 | 1.0 | 1.0 | 2.0 | 4.1×10$^{-4}$± 5.5×10$^{-5}$ |

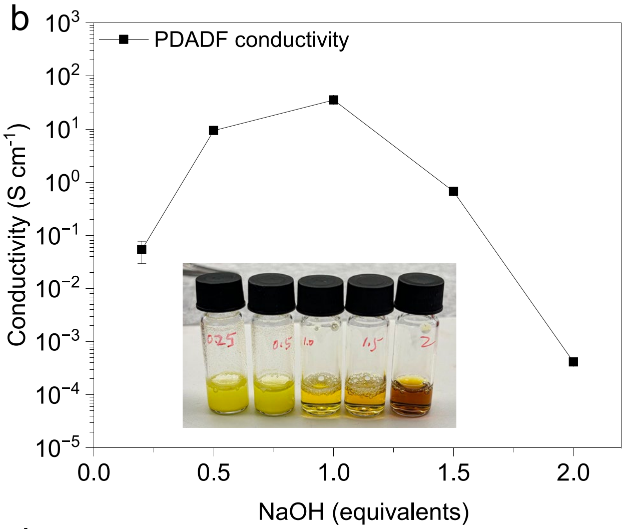


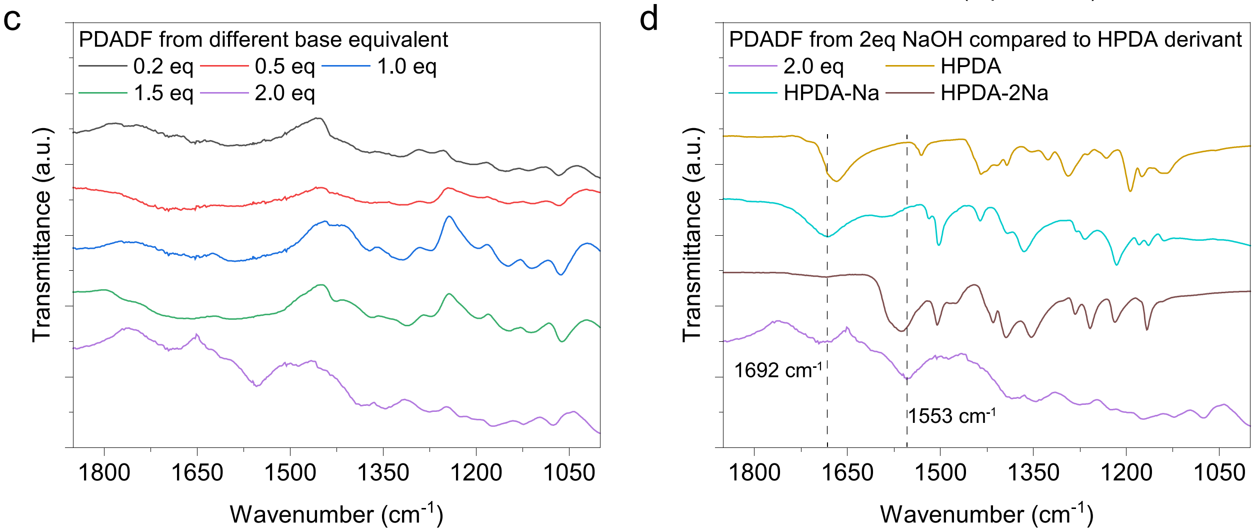


**Figure S16.** Polymerization with different equivalents of NaOH. (a) Polymerization scheme. (b) Electrical conductivity as a function of NaOH equivalents used during polymerization. (c) Offset FTIR spectra of polymers obtained from the polymerization scheme in (a). (d) Offset FTIR spectra of PDADF from 2 eq NaOH and HPDA derivatives. The highest electrical conductivity is reached for 1 eq NaOH. Similarities in the FTIR spectra for the samples up to 1.5 eq NaOH indicate that the polymers have a comparable defective structure. We tentatively attribute the lower electrical conductivity at NaOH < 1 eq to a lower degree of polymerization originating from the lower solubility of TMQ-PA. The use of higher equivalents of NaOH (i.e., 2 eq) yields a polymer with a higher content of opened lactone moieties, which is likely responsible for the lower electrical conductivity. Moreover, 2 eq leads to a more pronounced peak around 1692 cm$^{-1}$ and 1553 cm$^{-1}$, corresponding to COOH and asymmetric COO$^{-}$ vibrations, respectively. These features are qualitatively similar to those observed in the FTIR spectra of fully ring-opened HPDA derivatives HPDA-Na and HPDA-2Na, suggesting a higher content of opened lactone moieties when 2 eq of NaOH are used.

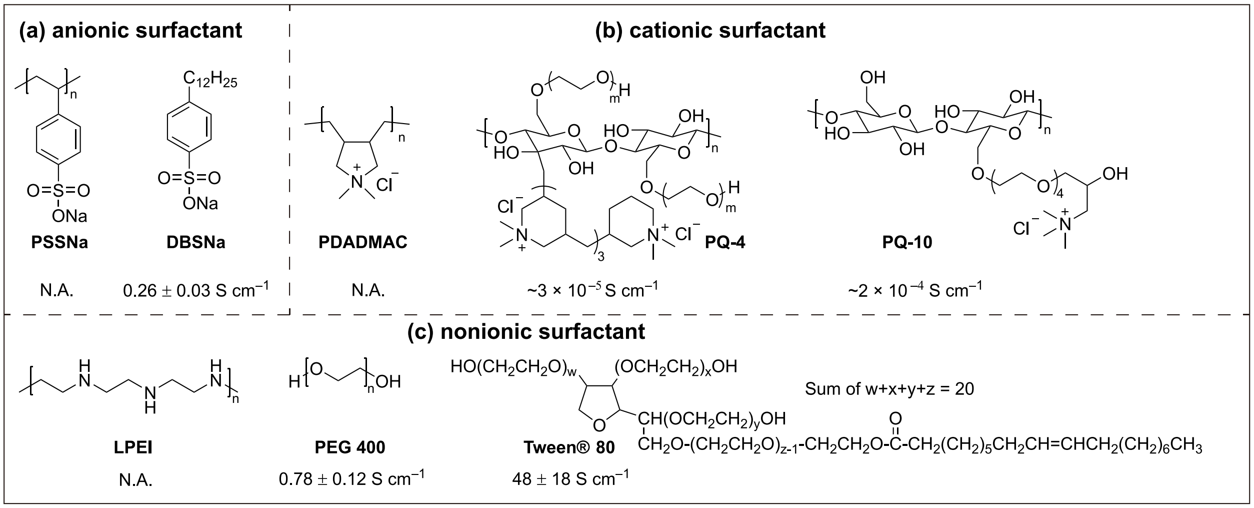


**Figure S17.** Different types of surfactants were added to the PDADF water dispersion to improve the electrical conductivity of the drop-cast films. (a) The use of anionic surfactants, both small molecules and polymer-based, was observed to diminish the electrical conductivity of the PDADF films. (b) The use of cationic surfactants like polyquaternium with varied functional groups has no discernible impact on the electrical conductivity. We attributed this to strong aggregation between PDADF and the cationic surfactants, causing non-uniform films and hindering any enhancement in electrical properties. (c) Nonionic surfactants such as linear PEI induced strong aggregation of PDADF, leading to a much-reduced electrical conductivity. Similarly, polyethylene glycol (PEG)-based surfactants demonstrated poor conductivity. In contrast, the nonionic surfactant Tween 80 (TW80) displayed improved conductivity. We propose that the optimal combination of hydrophilic glycol chains and hydrophobic groups in TW80 results in the formation of well-defined micelles in conjunction with PDADF, contributing to enhanced conductivity.

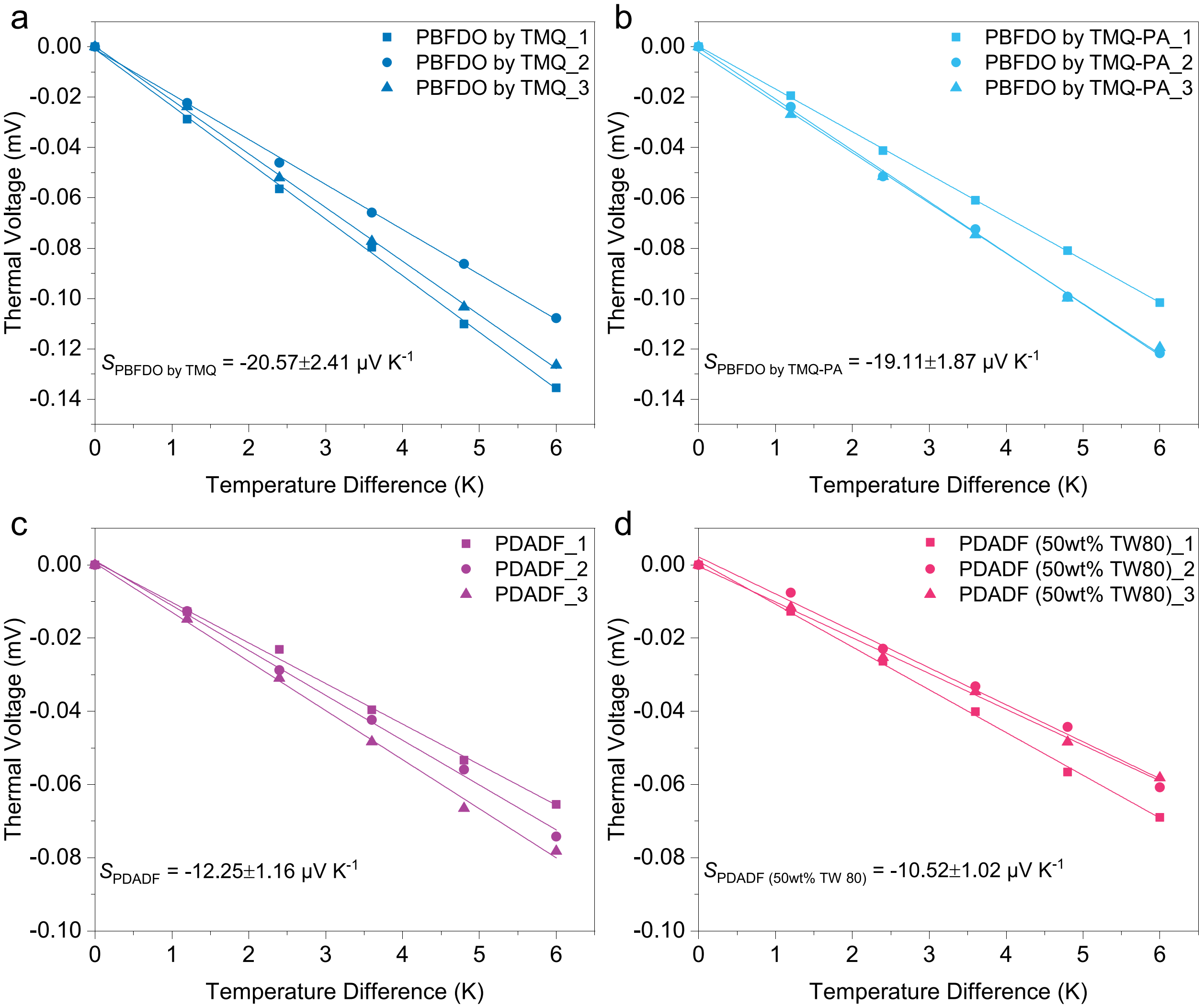


**Figure S18.** Seebeck coefficient measurements of PBFDO, PDADF, and PDADF (50 wt% TW80). The negative value indicates the n-type character of these polymers.

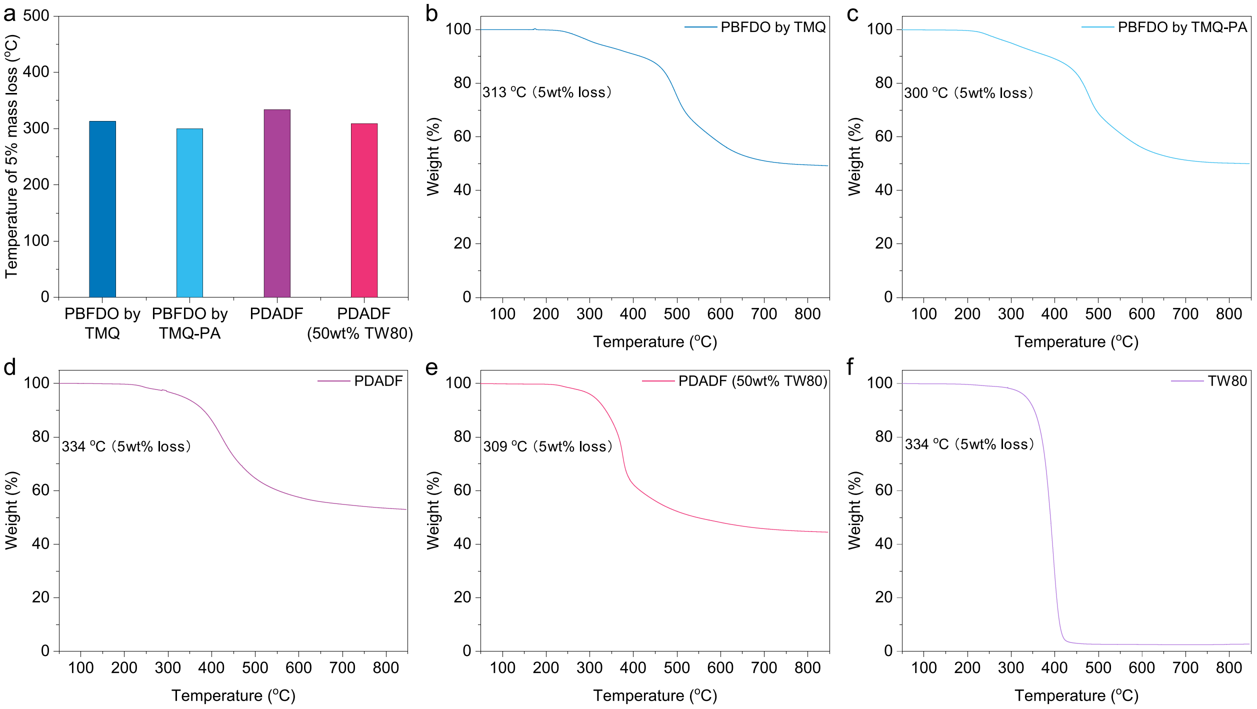


**Figure S19.** Thermogravimetric analysis (TGA) of PBFDO, PDADF, PDADF (50 wt% TW80), and TW80. (a) Summary of 5% weight loss of polymers as a function of temperature. (b) TGA of PBFDO by TMQ. (c) TGA of PBFDO by TMQ-PA. (d) TGA of PDADF. (e) TGA of PDADF (50 wt% TW80). (f) TGA of surfactant TW80.

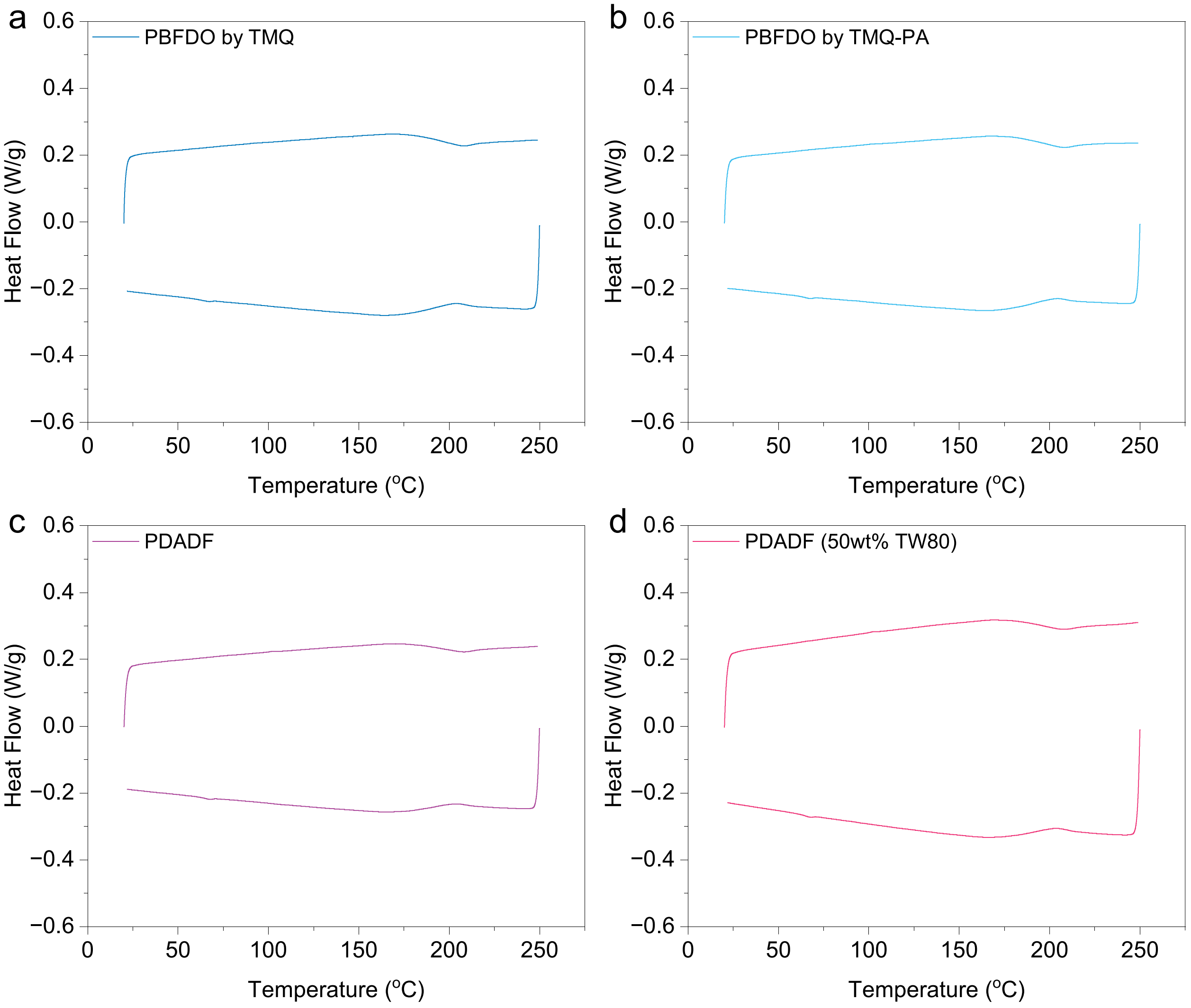


**Figure S20.** Differential scanning calorimetry (DSC) curves of polymers under nitrogen flow at a heating/cooling rate of 10/10 °C $min^{-1}$. All curves exhibit no obvious exothermic or endothermic features in the range of 25 ~ 250 °C.

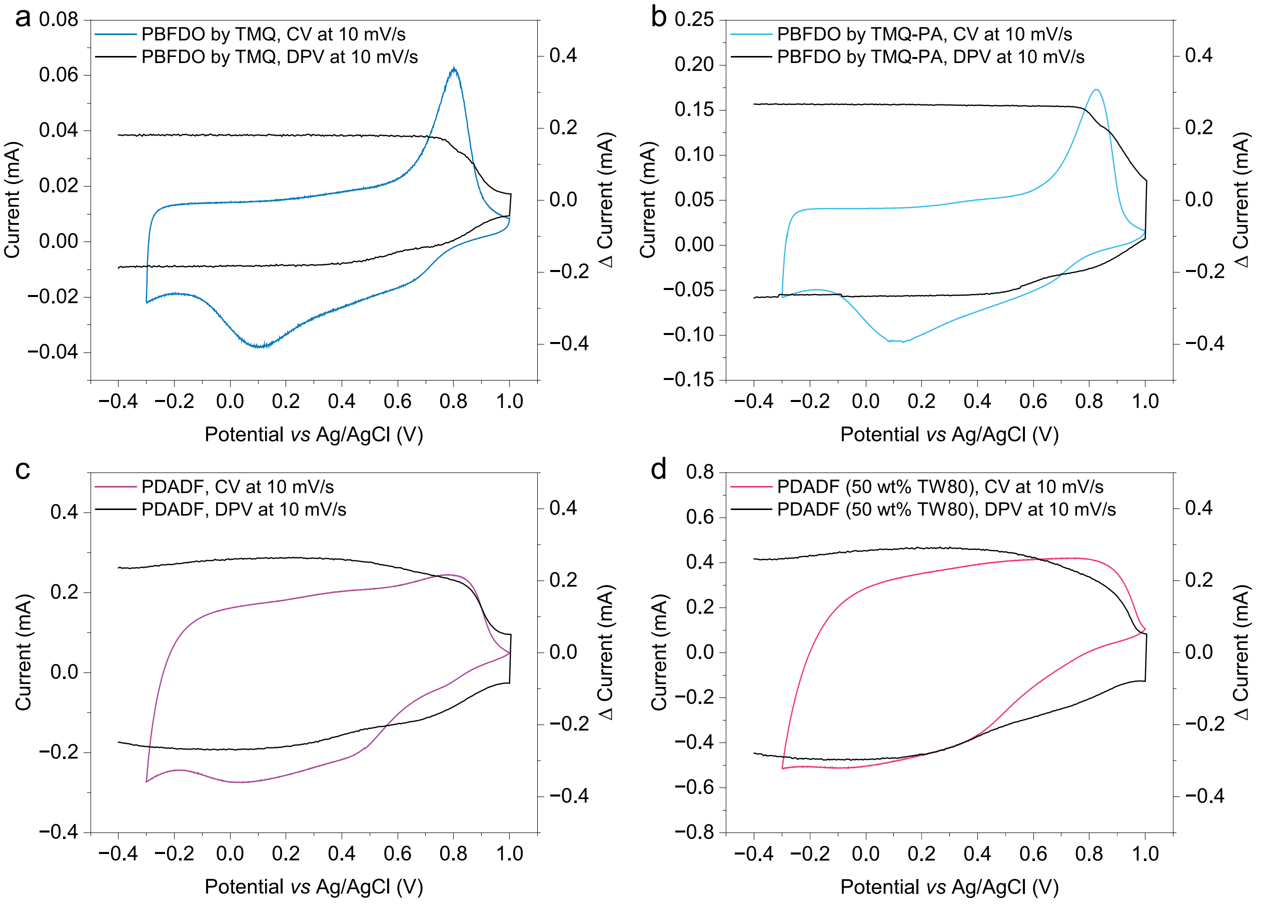


**Figure S21.** Cyclic voltammetry (CV) and differential pulse voltammetry (DPV) of drop-cast films measured with 0.2 M tetrabutylammonium bis-trifluoromethanesulfonimidate (TBA-TFSI) in propylene carbonate. (a) PBFDO synthesized using TMQ, (b) PBFDO synthesized using TMQ-PA. (c) PDADF. (d) PDADF (50 wt%, TW80).

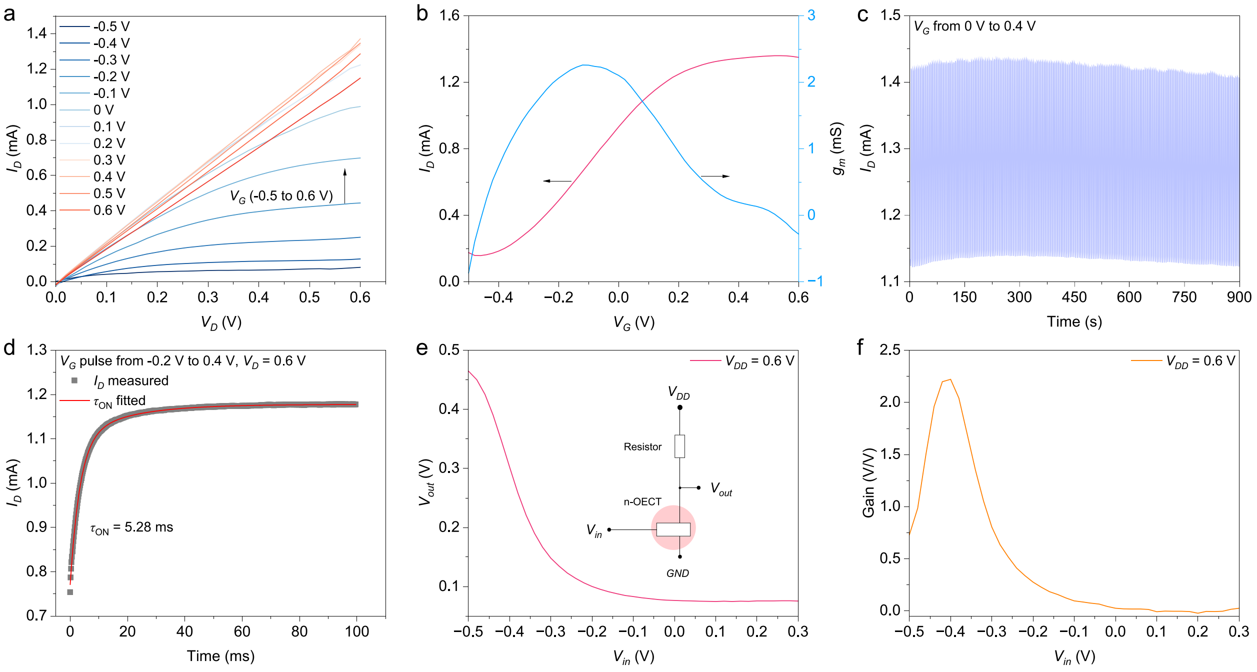


**Figure S22**. OECTs and OECT-based inverters performance. (a) Output and (b) transfer curves of PDADF-based OECTs. (c) Stability of PDADF-based OECTs under 0-0.4 V gate voltage pulses. (d) Transient response of PDADF-based OECT. (e) Typical voltage transfer characteristics and (f) voltage gains of the PDADF-based inverters. The PDADF films were cast and patterned as the n-type channel active material (width of 100 µm and length of 12 µm). An Ag/AgCl pellet was used as the gate electrode together with a 0.1 M NaCl aqueous electrolyte. The measured output and transfer characteristics are consistent with those expected for a partially doped conductive polymer. The max transconductance ($g_m$) reaches 2.26 mS at a gate voltage ($V_G$) of -0.1 V and source-drain voltage ($V_D$) of 0.6 V. No obvious degradation of the drain current ($I_D$) was observed when pulsing the $V_G$ between 0 V and 0.4 V for 15 min. Additionally, the transient response shows $\tau_{ON}$ = 5.28 ms. To further demonstrate PDADF-OECT applicability, we fabricated an inverter by connecting a 4 kΩ resistor in series with the PDADF-OECT. The resulting inverter operates at supply voltages ($V_{DD}$) of 0.6 V with a voltage gain over 2.2 V $V^{-1}$.

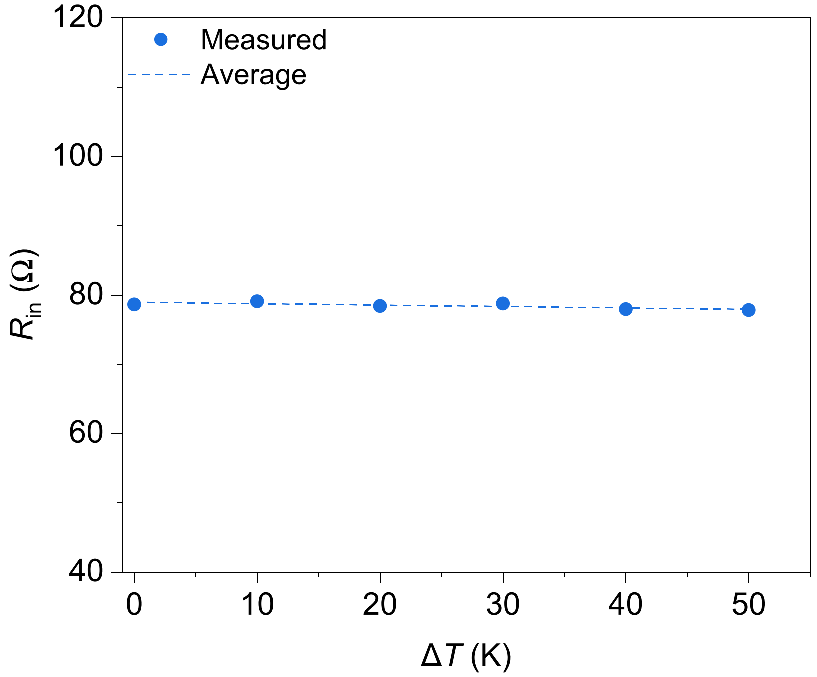


**Figure S23.** Internal resistance of one p-n pair TEG combination of PDADF (50 wt% TW80) and PEDOT:PSS. The result shows the stable internal resistance of TEG during the measurement with a mean value of 78 ohms.

**Table S3.** Comparison of in-plane geometry polymer thermoelectric generator: thermoelectric leg materials, thermos module units (one unit = a pair of p and n legs), working temperature difference ($\Delta T$), power output ($P_{output}$), and power output per p-n pair.

| n-leg material | p-leg material | p-n pair | $\Delta T$ (K) | $P_{output}$ (nW) | $P_{output}$ (per p-n pair, nW) | Reference |
|---|---|---|---|---|---|---|
| PDADF (50 wt% TW80) | PEDOT:PSS (gold electrodes) | 1 | 10 | 0.64 | 0.64 | This work |
| | | | 20 | 2.48 | 2.48 | |
| | | | 30 | 5.45 | 5.45 | |
| | | | 40 | 9.10 | 9.10 | |
| | | | 50 | 14.70 | 14.70 | |
| $BBL:PEI_{lin}$ | PEDOT:PSS (gold electrodes) | 1 | 5 | 0.51 | 0.51 | 6 |
| | | | 10 | 2.08 | 2.08 | |
| | | | 20 | 8.26 | 8.26 | |
| | | | 30 | 18.71 | 18.71 | |
| | | | 40 | 33.88 | 33.88 | |
| | | | 50 | 55.34 | 55.34 | |
| FBDPPV:TAM | PEDOT:PSS (gold electrodes) | 3 | 2.5 | 0.18 | 0.06 | 15 |
| | | | 4.3 | 0.51 | 0.17 | |
| | | | 9.8 | 2.75 | 0.92 | |
| | | | 17.5 | 9.24 | 3.08 | |
| | | | 27.8 | 23.75 | 8.25 | |
| | | | 46.5 | 77.02 | 25.67 | |
| TBDOPV-T-518:*N*-DMBI | TBDOPV-T-518:$FeCl_3$ | 3 | 9.4 | 20.0 | 6.7 | 16 |
| | | | 16.4 | 59.7 | 19.9 | |
| | | | 26.3 | 144 | 48.2 | |
| | | | 45.1 | 403 | 134 | |
| silver connection | PEDOT:PSS | 1 | 65 | 0.057 | 0.057 | 17 |
| silver connection | PEDOT:PSS | 5 | 75.2 | 12.29 | 2.46 | 18 |
| silver connection | PEDOT:PSS | 385 | 50 | 4000 | 10.39 | 19 |
| | | 1650 | 100 | 50000 | 30.30 | |
| gold connection | PEDOT:PSS | 10 | 10 | 2.6 | 0.26 | 20 |
| | | | 20 | 11 | 1.10 | |
| | | | 30 | 25 | 2.50 | |

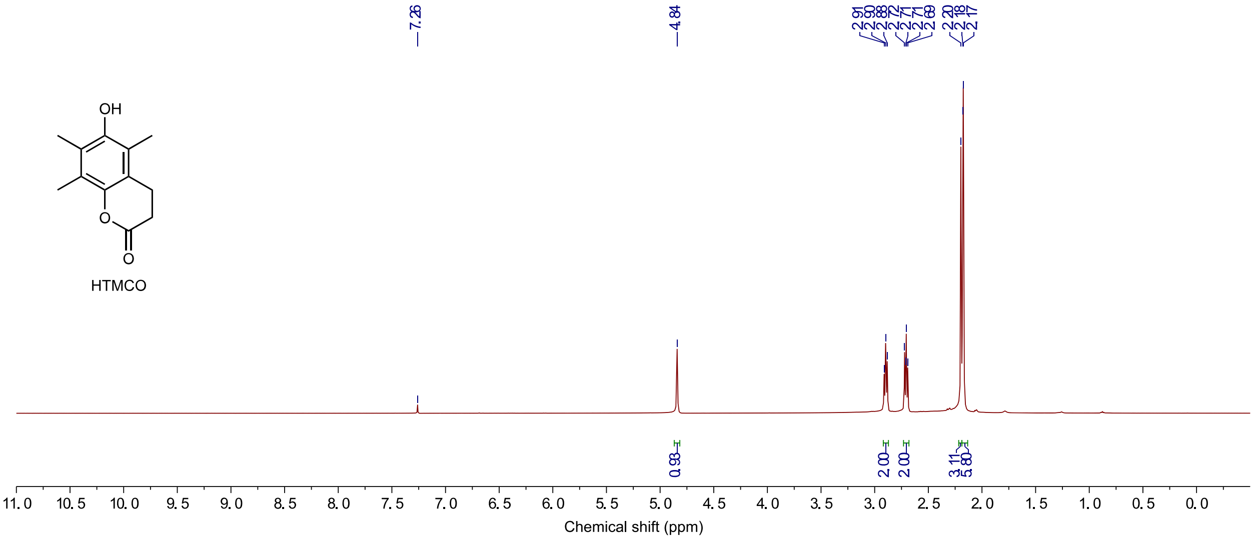


**Figure S24.** $^{1}$H NMR of HMTCO

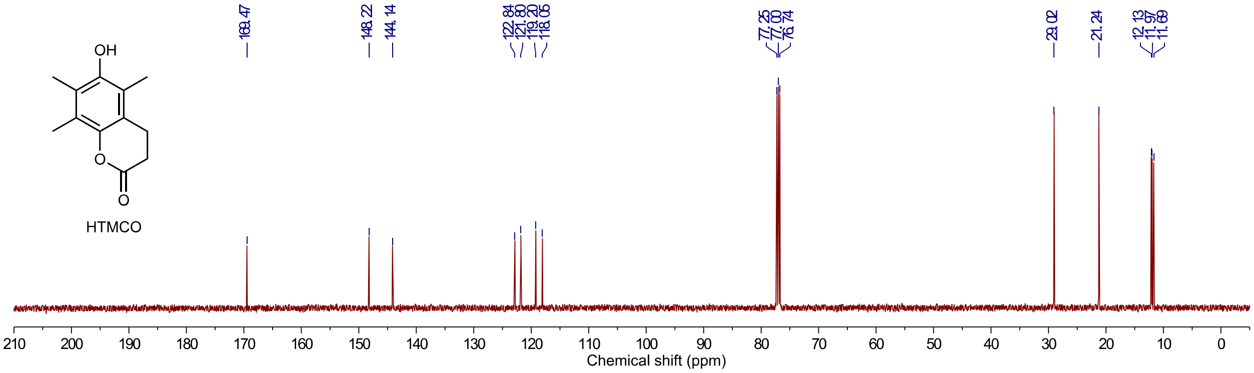


**Figure S25.** $^{13}$C NMR of HMTCO

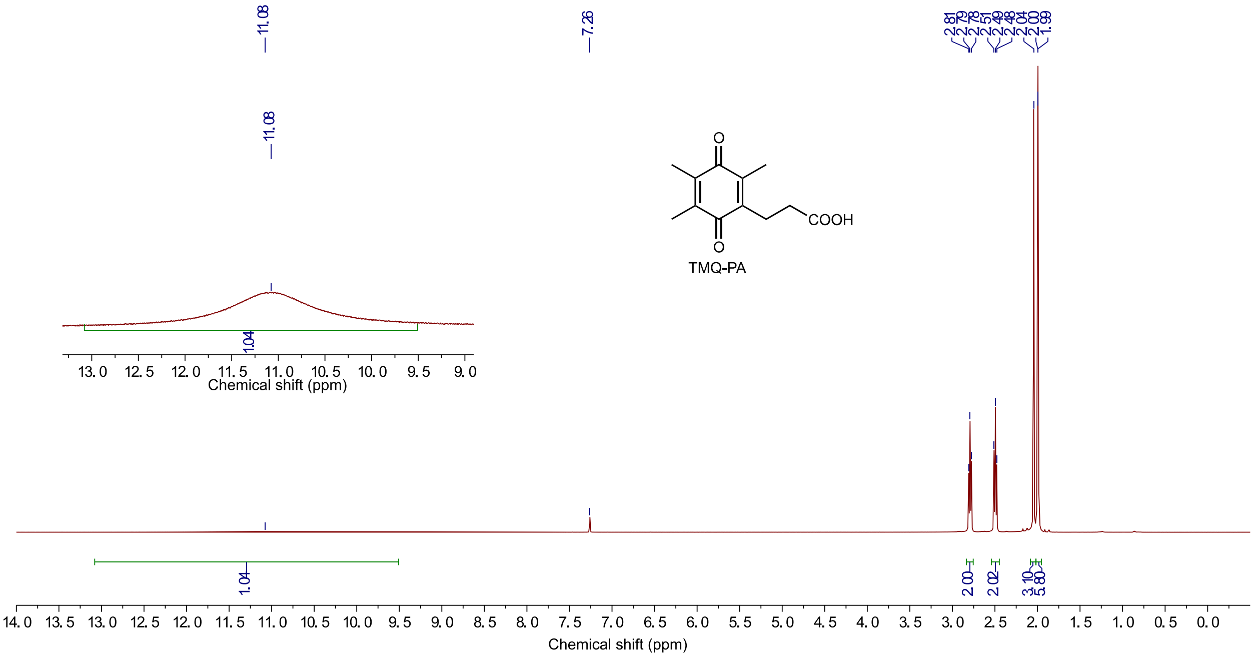


**Figure S26.** $^1$H NMR of TMQ-PA

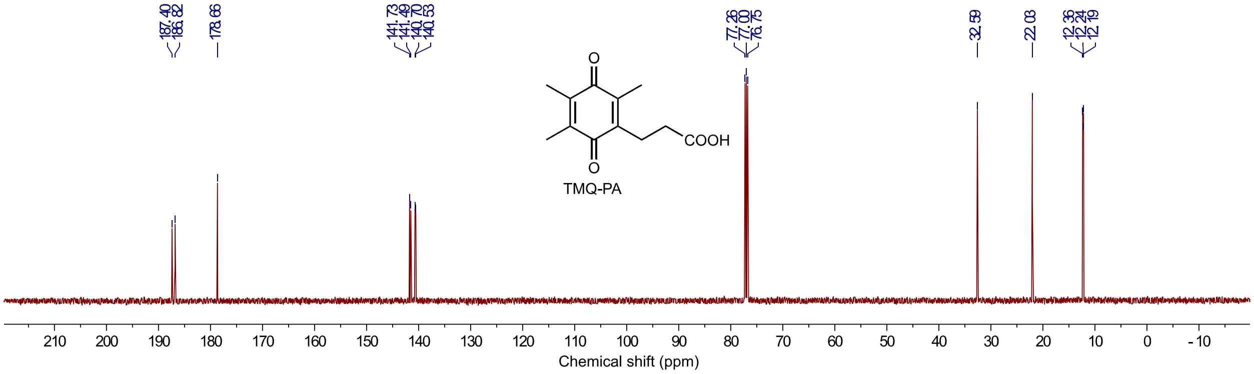


**Figure S27.** $^{13}$C NMR of TMQ-PA

# References


(1) Ong, W.; Yang, Y.; Cruciano, A. C.; McCarley, R. L. Redox-Triggered Contents Release from Liposomes. *J. Am. Chem. Soc.* **2008**, *130* (44), 14739–14744.

(2) Alsufyani, M.; Hallani, R. K.; Wang, S.; Xiao, M.; Ji, X.; Paulsen, B. D.; Xu, K.; Bristow, H.; Chen, H.; Chen, X.; Sirringhaus, H.; Rivnay, J.; Fabiano, S.; McCulloch, I. The Effect of Aromatic Ring Size in Electron Deficient Semiconducting Polymers for N-Type Organic Thermoelectrics. *J. Mater. Chem. C* **2020**, *8* (43), 15150–15157.

(3) Tang, H.; Liang, Y.; Liu, C.; Hu, Z.; Deng, Y.; Guo, H.; Yu, Z.; Song, A.; Zhao, H.; Zhao, D.; Zhang, Y.; Guo, X.; Pei, J.; Ma, Y.; Cao, Y.; Huang, F. A Solution-Processed n-Type Conducting Polymer with Ultrahigh Conductivity. *Nature.* **2022**, *611* (7935), 271–277.

(4) Wu, H.-Y.; Yang, C.-Y.; Li, Q.; Kolhe, N. B.; Strakosas, X.; Stoeckel, M.-A.; Wu, Z.; Jin, W.; Savvakis, M.; Kroon, R.; Tu, D.; Woo, H. Y.; Berggren, M.; Jenekhe, S. A.; Fabiano, S. Influence of Molecular Weight on the Organic Electrochemical Transistor Performance of Ladder-Type Conjugated Polymers. *Adv. Mater.* **2022**, *34* (4), 2106235.

(5) Liu, T.; Heimonen, J.; Zhang, Q.; Yang, C.-Y.; Huang, J.-D.; Wu, H.-Y.; Stoeckel, M.-A.; van der Pol, T. P. A.; Li, Y.; Jeong, S. Y.; Marks, A.; Wang, X.-Y.; Puttisong, Y.; Shimolo, A. Y.; Liu, X.; Zhang, S.; Li, Q.; Massetti, M.; Chen, W. M.; Woo, H. Y.; Pei, J.; McCulloch, I.; Gao, F.; Fahlman, M.; Kroon, R.; Fabiano, S. Ground-State Electron Transfer in All-Polymer Donor:Acceptor Blends Enables Aqueous Processing of Water-Insoluble Conjugated Polymers. *Nat. Commun.* **2023**, *14* (1), 8454.

(6) Yang, C.-Y.; Stoeckel, M.-A.; Ruoko, T.-P.; Wu, H.-Y.; Liu, X.; Kolhe, N. B.; Wu, Z.; Puttisong, Y.; Musumeci, C.; Massetti, M.; Sun, H.; Xu, K.; Tu, D.; Chen, W. M.; Woo, H. Y.; Fahlman, M.; Jenekhe, S. A.; Berggren, M.; Fabiano, S. A High-Conductivity n-Type Polymeric Ink for Printed Electronics. *Nat. Commun.* **2021**, *12* (1), 2354.

(7) Tang, H.; Liu, Z.; Tang, Y.; Du, Z.; Liang, Y.; Hu, Z.; Zhang, K.; Huang, F.; Cao, Y. Organic Diradicals Enabled N-Type Self-Doped Conjugated Polyelectrolyte with High Transparency and Enhanced Conductivity. *Giant.* **2021**, *6*, 100053.

(8) Lee, S.; Kim, Y.; Kim, D.; Jeong, D.; Kim, G.-U.; Kim, J.; Kim, B. J. Electron Transport Layers Based on Oligo(Ethylene Glycol)-Incorporated Polymers Enabling Reproducible Fabrication of High-Performance Organic Solar Cells. *Macromolecules.* **2021**, *54* (15), 7102–7112.

(9) Sharma, A.; Singh, S.; Song, X.; Rosas Villalva, D.; Troughton, J.; Corzo, D.; Toppare, L.; Gunbas, G.; Schroeder, B. C.; Baran, D. A Nonionic Alcohol Soluble Polymer Cathode Interlayer Enables Efficient Organic and Perovskite Solar Cells. *Chem. Mater.* **2021**, *33* (22), 8602–8611.

(10) Rahmanudin, A.; Marcial-Hernandez, R.; Zamhuri, A.; Walton, A. S.; Tate, D. J.; Khan, R. U.; Aphichatpanichakul, S.; Foster, A. B.; Broll, S.; Turner, M. L. Organic Semiconductors Processed from Synthesis-to-Device in Water. *Adv. Sci.* **2020**, *7* (21), 2002010.

(11) Jin, X.; Wang, Y.; Cheng, X.; Zhou, H.; Hu, L.; Zhou, Y.; Chen, L.; Chen, Y. Fluorine-Induced Self-Doping and Spatial Conformation in Alcohol-Soluble Interlayers for Highly-Efficient Polymer Solar Cells. *J. Mater. Chem. A* **2018**, *6* (2), 423–433.

(12) Chen, Z.; Hu, Z.; Wu, Z.; Liu, X.; Jin, Y.; Xiao, M.; Huang, F.; Cao, Y. Counterion-Tunable n-Type Conjugated Polyelectrolytes for the Interface Engineering of Efficient Polymer Solar Cells. *J. Mater. Chem. A* **2017**, *5* (36), 19447–19455.

(13) Wu, Z.; Sun, C.; Dong, S.; Jiang, X.-F.; Wu, S.; Wu, H.; Yip, H.-L.; Huang, F.; Cao, Y. N-Type Water/Alcohol-Soluble Naphthalene Diimide-Based Conjugated Polymers for High-Performance Polymer Solar Cells. *J. Am. Chem. Soc.* **2016**, *138* (6), 2004–2013.

(14) Jeong, D.; Jo, I.-Y.; Lee, S.; Kim, J. H.; Kim, Y.; Kim, D.; Reynolds, J. R.; Yoon, M.-H.; Kim, B. J. High-Performance n-Type Organic Electrochemical Transistors Enabled by Aqueous Solution Processing of Amphiphilicity-Driven Polymer Assembly. *Adv. Funct. Mater.* **2022**, *32* (16), 2111950.
(15) Yang, C.-Y.; Ding, Y.-F.; Huang, D.; Wang, J.; Yao, Z.-F.; Huang, C.-X.; Lu, Y.; Un, H.-I.; Zhuang, F.-D.; Dou, J.-H.; Di, C.; Zhu, D.; Wang, J.-Y.; Lei, T.; Pei, J. A Thermally Activated and Highly Miscible Dopant for N-Type Organic Thermoelectrics. *Nat. Commun.* **2020**, *11* (1), 3292.
(16) Yu, Z.-D.; Lu, Y.; Wang, Z.-Y.; Un, H.-I.; Zelewski, S. J.; Cui, Y.; You, H.-Y.; Liu, Y.; Xie, K.-F.; Yao, Z.-F.; He, Y.-C.; Wang, J.-Y.; Hu, W.-B.; Sirringhaus, H.; Pei, J. High N-Type and p-Type Conductivities and Power Factors Achieved in a Single Conjugated Polymer. *Sci. Adv.* **2023**, *9* (8), eadf3495.
(17) Søndergaard, R. R.; Hösel, M.; Espinosa, N.; Jørgensen, M.; Krebs, F. C. Practical Evaluation of Organic Polymer Thermoelectrics by Large-Area R2R Processing on Flexible Substrates. *Energy Sci. Eng.* **2013**, *1* (2), 81–88.
(18) Du, Y.; Cai, K.; Chen, S.; Wang, H.; Shen, S. Z.; Donelson, R.; Lin, T. Thermoelectric Fabrics: Toward Power Generating Clothing. *Sci. Rep.* **2015**, *5* (1), 6411.
(19) Wei, Q.; Mukaida, M.; Kirihara, K.; Naitoh, Y.; Ishida, T. Polymer Thermoelectric Modules Screen-Printed on Paper. *RSC Adv.* **2014**, *4* (54), 28802–28806.
(20) Kim, N.; Lienemann, S.; Petsagkourakis, I.; Alemu Mengistie, D.; Kee, S.; Ederth, T.; Gueskine, V.; Leclère, P.; Lazzaroni, R.; Crispin, X.; Tybrandt, K. Elastic Conducting Polymer Composites in Thermoelectric Modules. *Nat. Commun.* **2020**, *11* (1), 1424.
